# A highly attenuated vaccinia virus strain LC16m8-based vaccine for severe fever with thrombocytopenia syndrome

**DOI:** 10.1371/journal.ppat.1008859

**Published:** 2021-02-03

**Authors:** Tomoki Yoshikawa, Satoshi Taniguchi, Hirofumi Kato, Naoko Iwata-Yoshikawa, Hideki Tani, Takeshi Kurosu, Hikaru Fujii, Natsumi Omura, Miho Shibamura, Shumpei Watanabe, Kazutaka Egawa, Takuya Inagaki, Satoko Sugimoto, Supranee Phanthanawiboon, Shizuko Harada, Souichi Yamada, Shuetsu Fukushi, Shigeru Morikawa, Noriyo Nagata, Masayuki Shimojima, Masayuki Saijo

**Affiliations:** 1 Department of Virology 1, National Institute of Infectious Diseases, Musashimurayama-shi, Tokyo, Japan; 2 Department of Pathology, National Institute of Infectious Diseases, Musashimurayama-shi, Tokyo, Japan; 3 Department of Virology, Toyama Institute of Health, Imizu-shi, Toyama, Japan; 4 Department of Microbiology, Faculty of Veterinary Medicine, Okayama University of Science, Imabari-shi, Ehime, Japan; University of Texas Medical Branch / Galveston National Laboratory, UNITED STATES

## Abstract

Severe fever with thrombocytopenia syndrome (SFTS) caused by a species Dabie bandavirus (formerly SFTS virus [SFTSV]) is an emerging hemorrhagic infectious disease with a high case-fatality rate. One of the best strategies for preventing SFTS is to develop a vaccine, which is expected to induce both humoral and cellular immunity. We applied a highly attenuated but still immunogenic vaccinia virus strain LC16m8 (m8) as a recombinant vaccine for SFTS. Recombinant m8s expressing SFTSV nucleoprotein (m8-N), envelope glycoprotein precursor (m8-GPC), and both N and GPC (m8-N+GPC) in the infected cells were generated. Both m8-GPC- and m8-N+GPC-infected cells were confirmed to produce SFTSV-like-particles (VLP) *in vitro*, and the N was incorporated in the VLP produced by the infection of cells with m8-N+GPC. Specific antibodies to SFTSV were induced in mice inoculated with each of the recombinant m8s, and the mice were fully protected from lethal challenge with SFTSV at both 10^3^ TCID_50_ and 10^5^ TCID_50_. In mice that had been immunized with vaccinia virus strain Lister in advance of m8-based SFTSV vaccine inoculation, protective immunity against the SFTSV challenge was also conferred. The pathological analysis revealed that mice immunized with m8-GPC or m8-N+GPC did not show any histopathological changes without any viral antigen-positive cells, whereas the control mice showed focal necrosis with inflammatory infiltration with SFTSV antigen-positive cells in tissues after SFTSV challenge. The passive serum transfer experiments revealed that sera collected from mice inoculated with m8-GPC or m8-N+GPC but not with m8-N conferred protective immunity against lethal SFTSV challenge in naïve mice. On the other hand, the depletion of CD8-positive cells *in vivo* did not abrogate the protective immunity conferred by m8-based SFTSV vaccines. Based on these results, the recombinant m8-GPC and m8-N+GPC were considered promising vaccine candidates for SFTS.

## Author summary

Severe fever with thrombocytopenia syndrome (SFTS) is an emerging viral hemorrhagic fever with a high case-fatality rate (approximately 5% to >40%). Indigenous SFTS has been reported in China, Japan, South Korea, and Vietnam. Thus, the development of an effective vaccine for SFTS is urgently needed. Vaccinia virus (VAC) was previously used as a vaccine for smallpox. Unfortunately, after these strains, the so-called second generation of VAC used during the eradication campaign was associated with severe adverse events, and the third generation of VAC strains such as LC16m8 (m8) and modified vaccinia Ankara (MVA) was established. m8 is confirmed to be highly attenuated while still maintaining immunogenicity. m8 is licensed for use in healthy people in Japan. At the present time, approximately 100,000 people have undergone vaccination with m8 without experiencing any severe postvaccine complications. At present, third-generation VAC strains are attractive for a recombinant vaccine vector, especially for viral hemorrhagic infectious diseases, such as Ebola virus disease, Lassa fever, Crimean-Congo hemorrhagic fever, and SFTS. We investigated the practicality of an m8-based recombinant vaccine for SFTS as well as other promising recombinant VAC-based vaccines for viral hemorrhagic infectious diseases.

## Introduction

Severe fever with thrombocytopenia syndrome (SFTS) is an emerging viral hemorrhagic fever with a high case-fatality rate (approximately 5 to over 40%) [1–6]. The clinical symptoms are in general fever, malaise, myalgia, nausea, vomiting, and diarrhea. The laboratory findings include leukocytopenia and thrombocytopenia in the total blood cell counts, and elevated serum levels of hepatic enzymes [1, 7–11]. The disease is caused by the species *Dabie bandavirus*, which has been called *SFTS virus* (SFTSV) and later on *Huaiyangshan banyangvirus* [12], a novel tick-borne virus in the order *Bunyavirales*, family *Phenuiviridae*, and genus *Bandavirus*. The viral genome consists of three negative-stranded RNA segments and encodes four genes: RNA-dependent RNA polymerase, envelope glycoproteins precursor (GPC), nucleoprotein (N), and nonstructural protein. Indigenous SFTS had been reported in China, Japan, South Korea, and Vietnam [1–3, 13–15]. Hence the development of an effective vaccine for SFTS is urgently needed. Thus far, it has been reported that a live recombinant vesicular stomatitis virus or a DNA vaccine expressing the SFTSV glycoproteins (GPs) originated from SFTSV GPC gene-elicited protective immunity against SFTSV in lethal mouse or ferret models [16, 17].

Vaccinia virus (VAC) was used as the smallpox vaccine, and smallpox vaccines produced by using a variety of VAC strains were used during the global smallpox eradication program led by the World Health Organization. Since smallpox eradication was declared in 1980, VAC has been used as a recombinant vaccine vector with an expectation of immunogenicity [18, 19]. However, since the VAC strains used as the vaccine vector in the beginning of the eradication campaign were mainly so-called second-generation smallpox vaccines that were associated with severe side effects, such as encephalitis, encephalopathy, conjunctivitis, progressive vaccinia, eczema vaccinatum and generalized fetal VAC infections [18, 20, 21], the application of VAC has not been popular. The VAC strain LC16m8 (m8), which is categorized as a third-generation smallpox vaccine, as well as the modified vaccinia Ankara (MVA), is confirmed to have a highly attenuated phenotype but to maintain immunogenicity to protect against other orthopoxvirus infections, such as monkeypox [22–24]. The significant difference in the characteristics between m8 and MVA is the replication capacity in vitro. LC16m8 can infect mammalian cells and produce progeny viruses in rabbit kidney-based cells such as primary rabbit kidney cells and RK13 cells, although the host range of cell types is restricted. On the other hand, MVA can infect mammalian cells but cannot replicate well in most mammalian cells [21]. m8 is licensed for use in healthy people in Japan, and approximately 100,000 people have been vaccinated with m8 thus far, with antibody response comparable to the first generation vaccine [25] and without experiencing severe postvaccine complications [25, 26]. This well-balanced safety and immunogenicity profile of m8 represents a significant advantage over the first- and second generations of VAC. In the present study, recombinant m8 strains that express SFTSV N (m8-N), GPC (m8-GPC), or both N and GPC (m8-N+GPC) (m8-based SFTSV vaccines) were generated as SFTS vaccine candidates. It is hypothesized that N and GPC contributed to cellular immunity and/or humoral immunity against SFTSV infection. The immunogenicity and protective efficacy of m8-based SFTSV vaccines against SFTSV were evaluated using a mouse model of lethal SFTS.

## Results

### Generation and characterization of recombinant m8 harboring SFTSV genes

**Fig 1:**
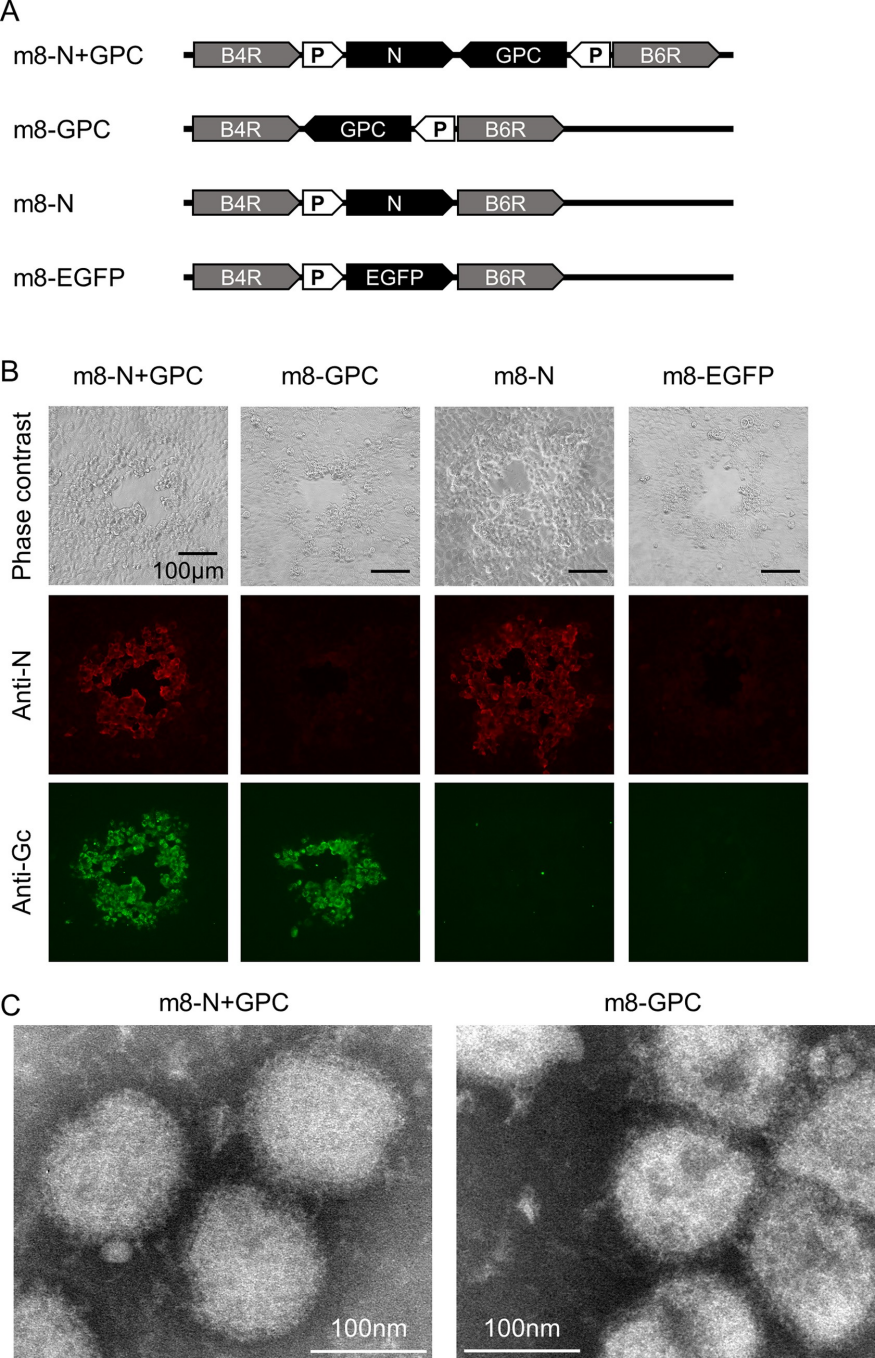
The construction, generation, and characterization of recombinant LC16m8 harboring SFTSV N, GPC, and both N and GPC, and EGFP genes. A schematic illustration of the site harboring EGFP, SFTSV genes in the genome in m8-EGFP, or m8-based SFTSV vaccines (A). The VAC B5R gene, flanked by the B4R and B6R genes, was substituted for the SFTSV gene. Synthetic vaccinia early/late promoter is indicated as P in the arrow-shaped box. The expression of SFTSV N and GPs was confirmed in the plaques of m8-based SFTSV vaccine-infected cells (B). The plaque formation was confirmed under phase-contrast microscopy (Phase contrast). SFTSV N expression was detected in an IFA using rabbit anti-SFTSV N (first antibody) and the DyLight 594-anti-rabbit IgG (second antibody) with visualization of N protein with the red fluorescent signal. The SFTSV GP expression was detected in an IFA using the mouse anti-SFTSV Gc (first antibody) and the DyLight 488-anti-mouse IgG (second antibody) with visualization of SFTSV GP with the green fluorescent signal. The bar in the image indicates a length of 100 μm. Purified VLP from the supernatant of the m8-N+GPC or m8-GPC-infected cell was negatively stained and observed under a transmission electron microscope (C). The supernatant was precipitated using PEG 6000, and the VLP was banded by ultracentrifugation at the interface of 20% and 60% (w/v) double-cushion sucrose. RK13 cells were infected with m8-N+GPC or m8-GPC at an MOI of 0.1 per cell, and the culture supernatant was collected at 3 DPI. The collected VLP in the culture supernatant was banded at the 20/60% interface and then negatively stained with 2% phosphotungstic acid. The bar in the image indicates the length of 100 nm. The morphology of SFTS VLP from the m8-N+GPC-infected cells and from that of m8-GPC was indistinguishable.

**Fig 2:**
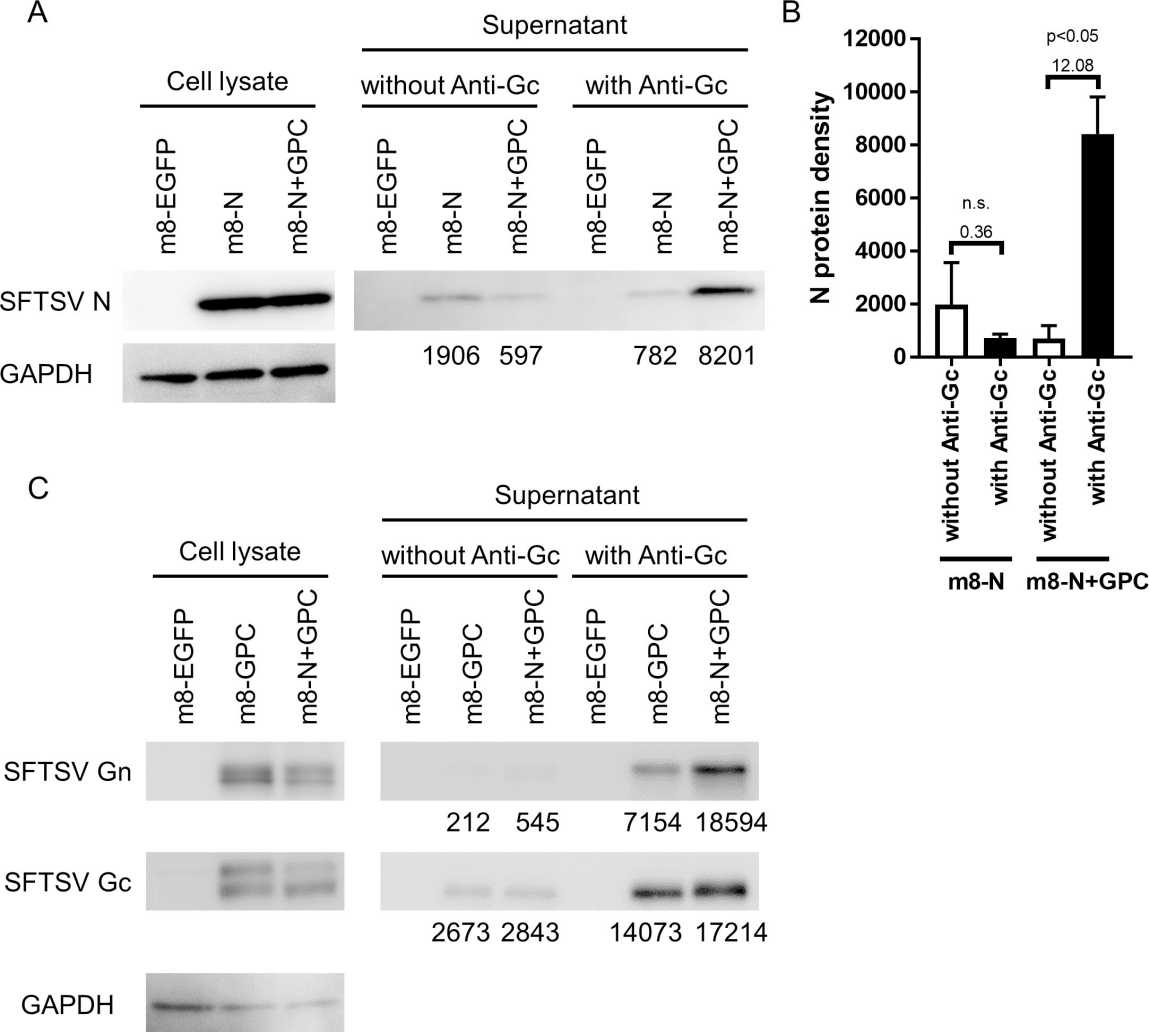
Incorporation of SFTSV N, Gn and Gc proteins into the VLP. SFTSV N protein incorporation into the VLP was evaluated (A). One representative out of three independent experiments is shown, and the remaining two blots are shown in S1 Fig. The VLP in the supernatant from the RK13 cells infected with m8-EGFP, m8-N, or m8-N+GPC was reacted with or without anti-SFTSV Gc mAb (clone C6C1) and then precipitated with anti-mouse IgG magnetic beads. The precipitated magnetic beads were then resolved on SDS-PAGE and analyzed by western blotting using rabbit anti-SFTSV N polyclonal antibody (SFTSV N), and the calculated N protein density is shown at the bottom of the blot. The N protein and a housekeeping gene, GAPDH protein expression (GAPDH), in RK13 cell lysates infected with m8-EGFP, m8-N, or m8-N+GPC are shown as a positive control. Accumulation of N protein with or without anti-Gc mAb for immunoprecipitation was semi-quantitatively analyzed (B). The calculated N protein density on blots from three independent experiments was plotted. SFTSV Gn and Gc protein incorporation into the VLP was evaluated as well as the N protein incorporation (C). The VLP in the supernatant of m8-EGFP, m8-GPC, or m8-N+GPC-infected RK13 cells was pulled down with anti-SFTSV Gc mAb and anti-mouse IgG magnetic beads. The accumulation of Gn and Gc proteins was confirmed by using rabbit anti-SFTSV Gn (SFTSV Gn) and Gc (SFTSV Gc) polyclonal antibody, respectively, are shown. The calculated Gn and Gc protein density are also described at the bottom of the blot. The Gn, Gc, and GAPDH protein expressions in RK13 cell lysates infected with m8-EGFP, m8-GCP, or m8-N+GPC are shown as a positive control. A Kruskal-Wallis test with Dunn’s multiple-comparison test was used to determine the level of statistical significance between the N protein density with and that without adding the anti-SFTSV Gc mAb. The calculated p-values and fold-change of the mean from without anti-Gc and anti-Gc are shown above the compared groups. n.s: not significant.

The final recombinant m8-based SFTSV vaccines harbored the SFTSV gene expression cassette in the flanking region between the B4R and B6R genes in place of the B5R gene (Fig 1A). The SFTSV N and Gc were detected in the cells infected with m8-N and m8-GPC, respectively, whereas both N and Gc were detected in the cells infected with m8-N+GPC (Fig 1B). The morphologies of the plaques induced by each of the recombinant m8s were similar. The plaques of the cells infected with m8-N and m8-GPC were positive only for the expression of the SFTSV N and GP, respectively. The plaques formed in the cells infected with m8-N+GPC showed a positive reaction in both N and GP expression. VLP production in the m8-GPC or m8-N+GPC-infected cells was evaluated by electron microscopy (Fig 1C). The morphological characteristics of VLP were enveloped and spherical with approximate diameters of 100 nm and seemed similar to that of SFTSV. Next, the incorporation of the N, Gn and Gc proteins into the VLP, which was produced from cells infected with m8-N+GPC, was evaluated (Fig 2). When the VLP was pulled down in immunoprecipitation using anti-SFTSV Gc mAb, the amount of N protein recovered from cells infected with m8-N+GPC was more extensive than that of m8-N-infected cells in comparison to when no antibodies were used. On the other hand, the addition of the anti-SFTSV Gc antibody did not affect the recovery of the N protein from the m8-N-infected-cell supernatant, which did not contain the VLP (Fig 2A and S1 Fig). From three independent experiments, it was confirmed that the N protein’s density in the immunoprecipitation was significantly intensified when the N protein was recovered from the supernatant of the cells infected with m8-N+GPC by using anti-SFTSV Gc mAb in comparison with that by using no antibody (Fig 2B). In contrast, the N protein density of the N protein recovered from the supernatant of the cells infected with m8-N by using anti-SFTSV Gc antibodies was the same level as that by using no antibodies (Fig 2B). The level of the Gn and Gc proteins’ incorporation into the VLP was evaluated (Fig 2C). The densities of both Gn and Gc proteins were intensified when the VLP produced in m8-N+GPC- or m8-GPC-infected cells was pulled down in immunoprecipitation using anti-SFTSV Gc mAb. This result indicated that the m8-GPC- or m8-N+GPC-infected cells produced SFTSV-like particles, and that the glycoproteins or both N and glycoproteins were incorporated into the VLP.

### Immunogenicity of m8-based SFTSV vaccines in mice

**Fig 3:**
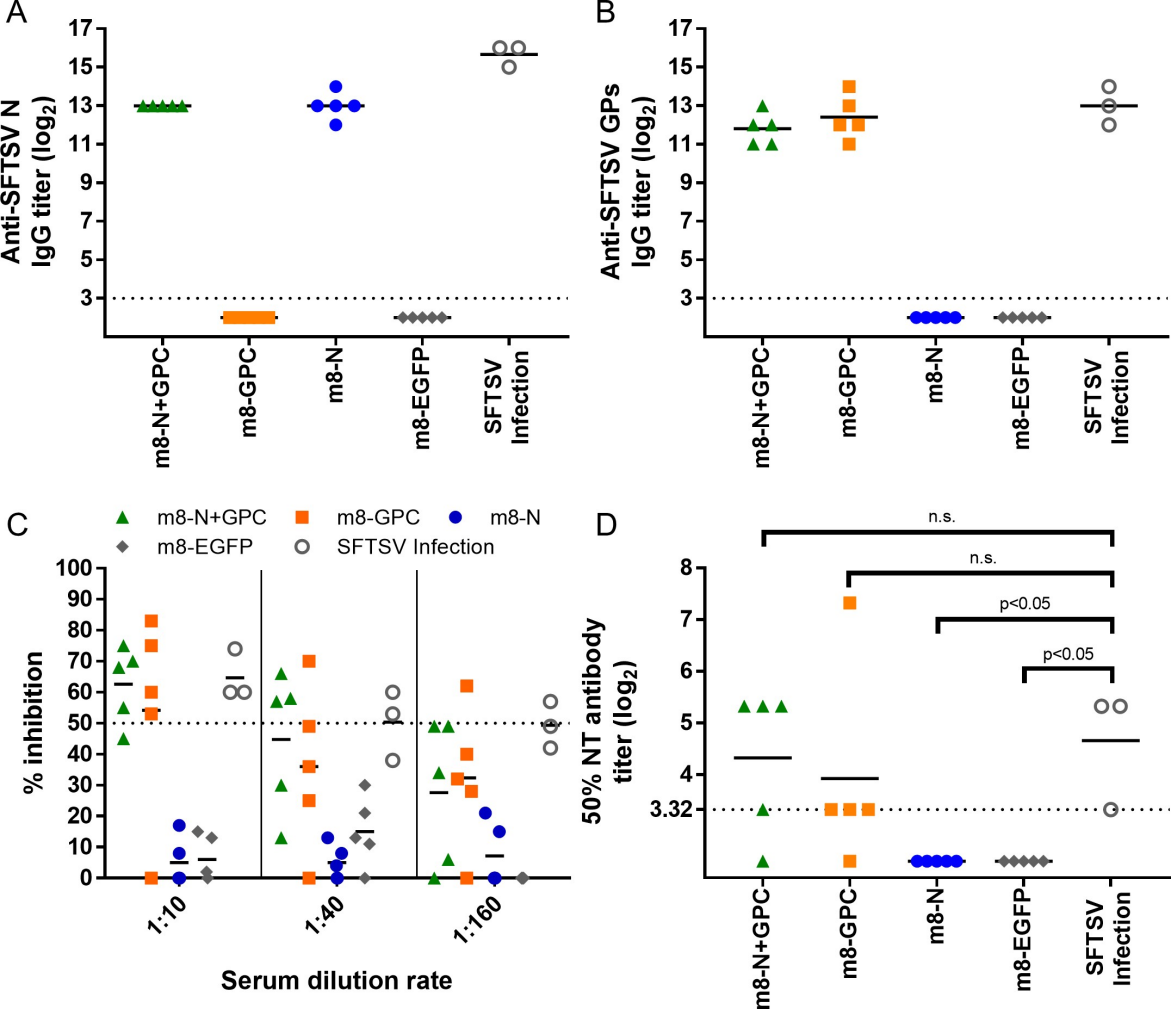
Immunogenicity of the m8-based SFTSV vaccines in mice. Naïve IFNAR-/- mice were subcutaneously inoculated twice at a 2-week interval with a dose of 1 × 10^6^ PFU of m8-EGFP, m8-N, m8-GPC or m8-N+GPC or subcutaneously infected with 10 TCID_50_ of SFTSV YG-1 (SFTSV infection). Two weeks after the second inoculation of mice with the recombinant m8s or 4 weeks after SFTSV infection, the mice were euthanized and sacrificed. Blood was collected to evaluate Anti-SFTSV N-specific (A) or GPs-specific (B) IgG production. Each dot represents the antibody titer. Horizontal lines represent the mean value. The horizontal dotted line at “2^3^” indicates the detection limit. The neutralizing antibodies to SFTSV were also measured (C) SFTSV YG-1 was mixed and incubated with 3 serial 4-fold dilutions (1/10, 1/40, or 1/160) of serum. The percent neutralization titer was determined to divide the number of foci by that of naïve mice. The 50% NT antibody titer (D) was determined from the result shown in Fig 3C. The horizontal dotted line at “2^3.32^”, which is equal to the real number 10, indicates the detection limit. A Kruskal-Wallis test with Dunn’s multiple-comparison test was used to determine the level of statistical significance. The calculated p-values are shown above the groups that were compared. n.s: not significant.

Groups (5 per group) of seven- to 8-week-old naïve Type I interferon alpha receptor-deficient (IFNAR-/-) mice were subcutaneously inoculated twice at a 2-week interval with either m8-based SFTSV vaccine or m8-EGFP as a negative control. None of the mice vaccinated with any of the m8-based SFTSV vaccines, including the negative control, developed observable clinical signs of illness, such as ruffled fur or pock formation at the site of inoculation. To evaluate the immunogenicity of m8-based SFTSV vaccines, sera were collected from IFNAR-/- mice and subjected to an IFA and neutralization test (NT). Five naïve IFNAR-/- mice were subcutaneously infected with 10 TCID_50_ of SFTSV YG-1 as a positive control. Since one of the five mice died at 13 days post-SFTSV infection, sera were collected from four mice at four weeks after infection. In addition, one of the sera collected from four SFTSV-infected mice was omitted from the subsequent analyses since the serum did not seroconvert (i.e., both IFA and NT results were negative). The specific IgG against SFTSV N or GPs in the sera were elicited by the infection of mice with m8-N, m8-GPC, or m8-N+GPC (Fig 3A and 3B). The NT antibodies to SFTSV were also elicited (Fig 3C). The 50% neutralization titers of the sera collected from m8-GPC-infected, m8-N+GPC-infected, and even SFTSV-infected mice were not significantly different (Fig 3D). Two mice inoculated with m8-N+GPC or m8-GPC were induced less than 10 of 50% NT antibody titer, although both mice induced specific IgG against SFTSV GPs. These results indicated that m8-based SFTSV vaccines induced humoral immunity, and the induction potency was comparable to that induced by the authentic SFTSV.

### Vaccine efficacy of m8-based SFTSV vaccines in mice

**Fig 4:**
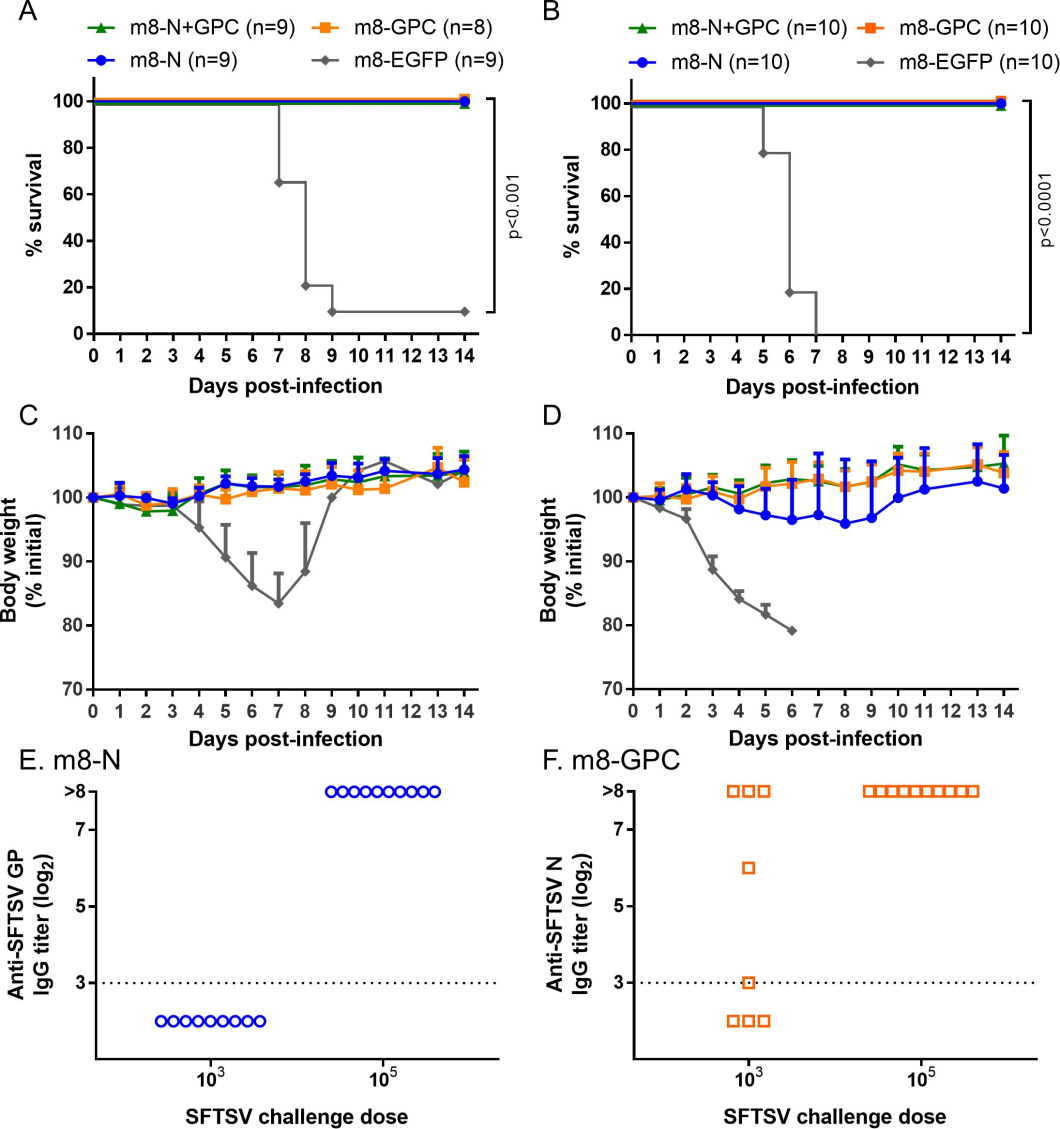
Survival, weight change, and seropositivity in m8-based SFTSV vaccine-inoculated mice followed by lethal SFTSV challenge. IFNAR-/- mice were subcutaneously inoculated twice at a 2-week interval with a dose of 1 × 10^6^ PFU of m8-EGFP, m8-N, m8-GPC or m8-N+GPC. Two weeks later, from the second inoculation of mice with m8-EGFP or m8-based SFTSV vaccines, the mice were subcutaneously challenged with 1 × 10^3^ (A, C) or 1 × 10^5^ (B, D) TCID_50_ of SFTSV YG-1. Survival (A, B) and the sequential change in percent weight from the initial (C, D) were followed daily for 2 weeks. The number of mice in a group is shown as “n” in the legend. The log-rank test was used to determine the level of statistical significance. The calculated p-values are shown beside the groups that were compared. Seropositivity in mice inoculated with m8-N or m8-GPC then infected with SFTSV was evaluated. Serum was collected from the m8-N (E) and m8-GPC-inoculated mice (F) at 2 weeks after 1 × 10^3^ or 1 × 10^5^ TCID_50_ of SFTSV infection for verifying whether seroconversion was induced by SFTSV infection or not. Specific IgG against SFTSV GPs and N, which should be induced by SFTSV infection, and which should never be induced by monovalent m8-N and m8-GPC, respectively, were measured. Each dot represents the antibody titer. The horizontal dotted line at 2^3^ indicates the detection limit.

Groups (8 to 10 per group) of six- to nine-week-old IFNAR-/- mice were subcutaneously inoculated twice at a two-week interval with each m8-based SFTSV vaccine. At two weeks after the second inoculation, the mice were subcutaneously challenged with 1 × 10^3^ or 1 × 10^5^ TCID_50_ of SFTSV YG-1. While almost all of the control mice died after the development of clinical signs, such as ruffled fur, hunched posture, and weight loss, all of the mice inoculated with either m8-GPC or m8-N+GPC survived without developing any obvious clinical signs during the two-week observation period after the SFTSV challenge (Fig 4A–4D). Furthermore, all the mice that had been inoculated with m8-N survived without developing any obvious clinical signs after SFTSV challenge with a dose of 1 × 10^3^ TCID_50_ during the two-week observation period (Fig 4A and 4C). On the other hand, the mice inoculated with m8-N survived but transiently developed clinical signs, when challenged with 1 × 10^5^ TCID_50_ of SFTSV (Fig 4B and 4D). To assess the immune response to N and GPs of SFTSV by evaluating seroconversion, sera collected from mice that survived two weeks after the SFTSV infection were subjected to an IFA to measure specific IgG against SFTSV N and GPs, which would not be induced by monovalent m8-GPC and m8-N, respectively. Since m8-N+GPC inoculated mice induced specific IgG against both SFTSV N and GPs, the sera of the m8-N+GPC inoculated mice were not subjected to the IFA. SFTSV GPs-specific IgG was not induced in the m8-N inoculated mice, while N-specific IgG was induced in five of eight m8-GPC-inoculated mice when challenged with 1 × 10^3^ TCID_50_ SFTSV (Fig 4E and 4F). On the other hand, all mice inoculated with m8-N or m8-GPC seroconverted after infection with 1 × 10^5^ TCID_50_ of SFTSV. These results indicated that the m8-based SFTSV vaccines conferred protection against a lethal SFTSV challenge. There was a difference in the degree of the efficacy between m8-N and m8-GPC including m8-N+GPC.

### Effect of preexisting immunity against VAC

**Fig 5:**
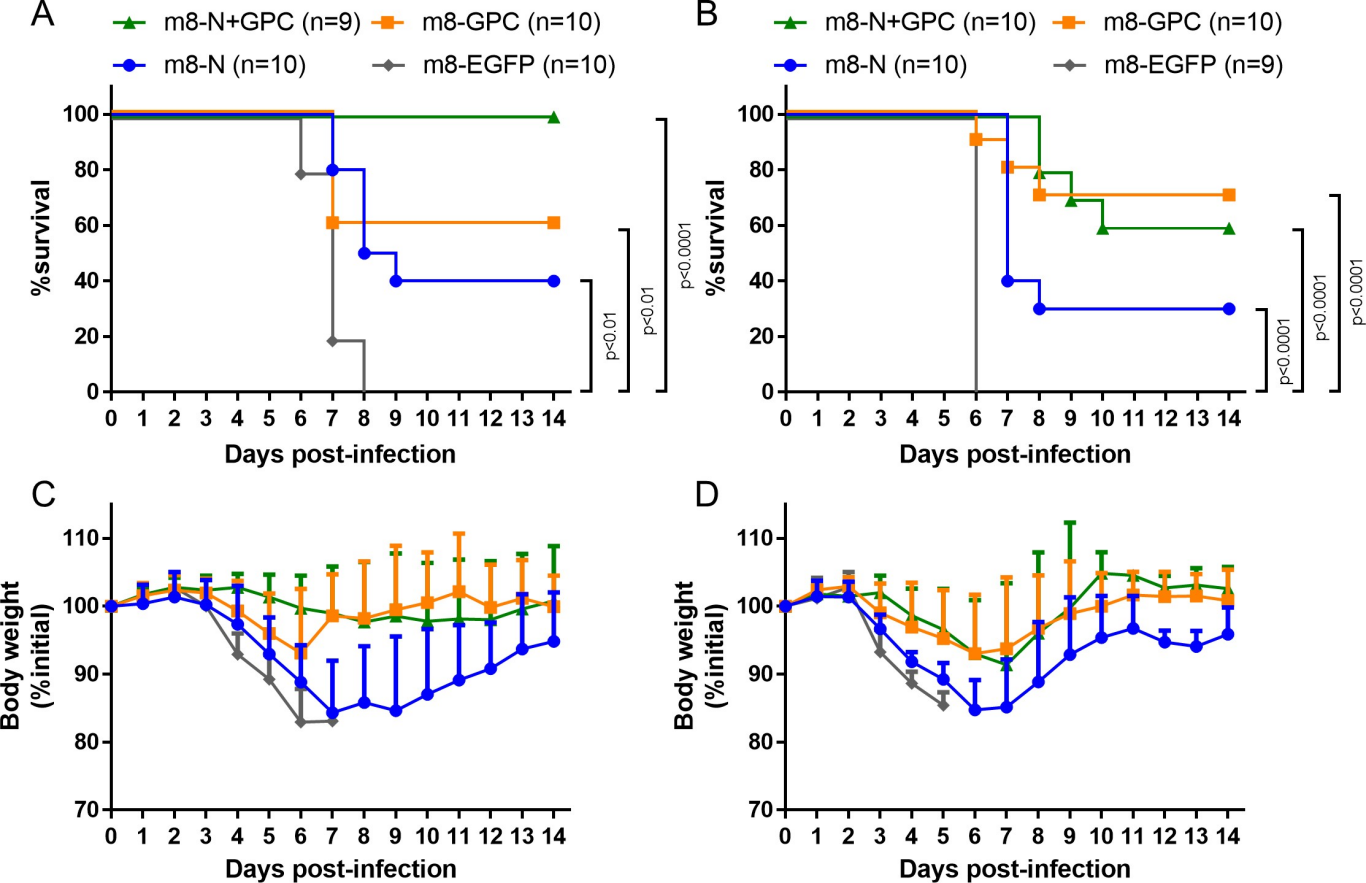
Survival and weight change in mice immunized with VAC before m8-based SFTSV vaccine inoculation. IFNAR-/- mice were inoculated twice at a 2-week interval with a dose of 1 × 10^6^ PFU of m8-EGFP, m8-N, m8-GPC or m8-N+GPC, 1 month after inoculation with 1 × 10^6^ PFU VAC strain Lister. Two weeks after the second inoculation of m8-EGFP, m8-N, m8-GPC, or m8-N+GPC, the mice were subcutaneously challenged with 1 × 10^3^ (A, C) or 1 × 10^5^ (B, D) TCID_50_ of SFTSV YG-1. Survival (A, B) and percent weight sequential change from the initial (C, D) were followed daily for 2 weeks. The number of mice used in a group is shown as “n” in the legend. The log-rank test with Bonferroni correction was used to determine the level of statistical significance. To adjust the significance level, the basic significance level was divided by the number of null hypotheses tested (i.e., 0.05/3 = 0.017). The calculated p-values are shown beside the groups that were compared.

To evaluate the impact of preexisting immunity to VAC on the protective immunity induced by m8-based SFTSV vaccines, IFNAR-/- mice were immunized with VAC and then inoculated with each m8-based SFTSV vaccine and challenged with SFTSV. The survival rate of the VAC-preimmunized mice infected with 1 × 10^3^ or 1 × 10^5^ TCID_50_ of SFTSV was improved by inoculation with m8-N, m8-GPC, or m8-N+GPC in comparison to control mice inoculated with m8-EGFP (Fig 5). However, the survival rates were lower than those of the mice that were not immunized with VAC Lister in advance of the vaccinations (Figs 4A and 4B, 5A and 5B). The survival rate of m8-N+GPC-inoculated mice was similar to that of m8-GPC-inoculated mice, whereas the survival rate of m8-N-inoculated mice was lower in comparison to m8-N+GPC- and m8-GPC-inoculated mice. The disease severity of the groups tended to show a similar trend to the survival rate. Although all the mice, even those inoculated with m8-N, m8-GPC, m8-N+GPC in advance, developed clinical symptoms after infection with 1 × 10^3^ TCID_50_ or 1 × 10^5^ TCID_50_ of SFTSV, the weight loss in the mice inoculated with m8-based SFTSV vaccines, especially those inoculated with m8-GPC or m8-N+GPC, was milder in comparison to mice inoculated with m8-EGFP (Fig 5C and 5D). The number of surviving mice decreased from ten to six and four to three in the m8-N+GPC-inoculated mice and the m8-N-inoculated mice, respectively, by increasing the challenging dose of SFTSV from 1 × 10^3^ to 1 × 10^5^ TCID_50_ (Fig 5A and 5B). Although the survival number in the m8-GPC-inoculated mice increased from six to seven, the increase might have been due to experimental fluctuations. This speculation seems to be supported by the trend of body weight change (Fig 5C and 5D). There were no differences in the body weight change trend between the mice inoculated with m8-GPC challenged with 1 × 10^3^ TCID_50_ of SFTSV and those challenged with 1 × 10^5^ TCID_50_ of SFTSV. Similar phenomena were also demonstrated between the mice inoculated with m8-N+GPC challenged with 1 × 10^3^ of TCID_50_ of SFTSV and those challenged with 1 × 10^5^ TCID_50_ of SFTSV. On the other hand, the trend of body weight change in m8-N-inoculated mice resembled that of m8-EGFP-inoculated mice, although the m8-N-inoculated mice survived partially (Fig 5C and 5D). LC16m8-GPC and m8-N+GPC were equally as effective as SFTSV vaccines. These results indicated that m8-based SFTSV vaccines conferred protection against lethal SFTSV challenge in mice, even when preexisting immunity to VAC was present.

### SFTSV infection dynamics in mice inoculated with m8-based SFTSV vaccines

**Fig 6:**
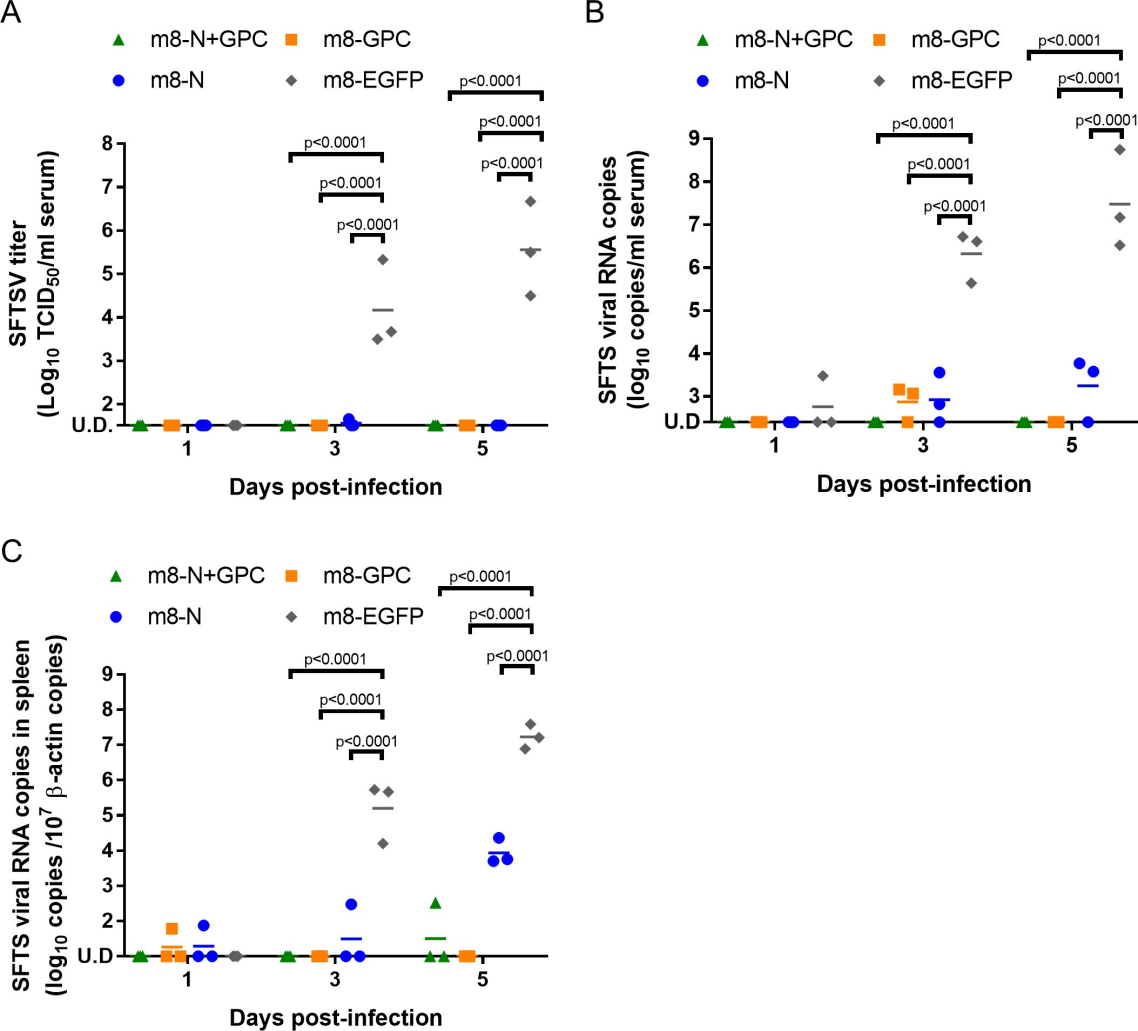
Infectious SFTSV and SFTSV genome levels and the histopathological changes in the tissue of m8-based vaccine-inoculated mice. IFNAR-/- mice were inoculated with m8-N, m8-GPC, m8-N+GPC, or m8-EGFP. At two weeks after the second inoculation, the mice were subcutaneously challenged with 1 × 10^5^ TCID_50_ of SFTSV YG-1. Three infected mice were sacrificed, and blood was collected at 1, 3, and 5 DPI. The sequential change in the infectious SFTSV titers in the sera was determined with a standard TCID_50_ assay (A). The SFTSV RNA copies were determined in the sera with qPCR (B) and in the spleen (C), which was normalized by 1 × 10^7^ copies of murine β-actin. A two-way ANOVA with Sidak’s multiple-comparison test was used to determine the level of statistical significance. The calculated p-values are shown above the groups that were compared. U.D., under the detection limit, which is 32 TCID_50_ per ml, 125 copies per ml of serum or 10 copies per 1 × 10^7^ copies of murine β-actin in spleen.

To investigate the efficacy of m8-based SFTSV vaccines in mice on spatial and temporal SFTSV infection dynamics, 6-week-old IFNAR-/- mice (three per group on each collection day) were subcutaneously inoculated twice at a two-week interval with m8-based SFTSV vaccines. Two weeks after the second inoculation, the mice were subcutaneously challenged with 1 × 10^5^ TCID_50_ of SFTSV YG-1, and the sera and tissues were collected at 1, 3, and 5 DPI. Sera collected from mice inoculated with m8-EGFP showed a high SFTSV titer (Fig 6A). In contrast, in sera from the mice inoculated with m8-N, m8-GPC, or m8-N+GPC, SFTSV was below the limit of detection or a tiny amount of the virus was detected (Fig 6A). SFTSV RNA was detected in the sera in one-third of the m8-EGFP inoculated mice at 1 DPI, and the copy number was drastically increased at 3 and 5 DPI (Fig 6B). The average viral RNA levels in 1 ml of serum were 1 × 10^6.3^ and 1 ×10^7.5^ copies at 3 and 5 DPI, respectively. On the other hand, the viral RNA level in sera of mice inoculated with m8-based SFTSV vaccines was significantly lower than that of the mice inoculated with m8-EGFP. However, the transition of the level differed among mice inoculated with m8-N, m8-GPC, and m8-N+GPC (Fig 6B). The SFTSV RNA was detected in m8-N-inoculated mice over 3–5 days, although the amount of viral RNA was small. In the sera of mice inoculated with m8-GPC, the viral RNA had been detected at 3 DPI but was below the limit of detection at 5 DPI. The viral RNA level was below the detection limit in the sera of the m8-N+GPC-inoculated mice not only at 3 DPI but also at 5 DPI. The sequential change of the viral RNA level in the spleen was also measured (Fig 6C). Although the amount of viral RNA and positive number was small, the viral RNA was detected in the spleens of the m8-GPC-inoculated mice and m8-N-inoculated mice at 1 DPI and was also detected in the spleen of the m8-N+GPC-inoculated mice at 3 DPI in one of 3 mice each. At 5 DPI, the viral RNA level in the spleens increased not only in the m8-EGFP-inoculated mice but also in those inoculated with m8-N. These results also indicated that seroconversion against N protein in the m8-GPC-inoculated mice occurred due to the SFTSV infection, which was supported by the detection of low-level viral RNA (Figs 4F and 6).

### Histopathological and immunohistochemical analysis

**Fig 7:**
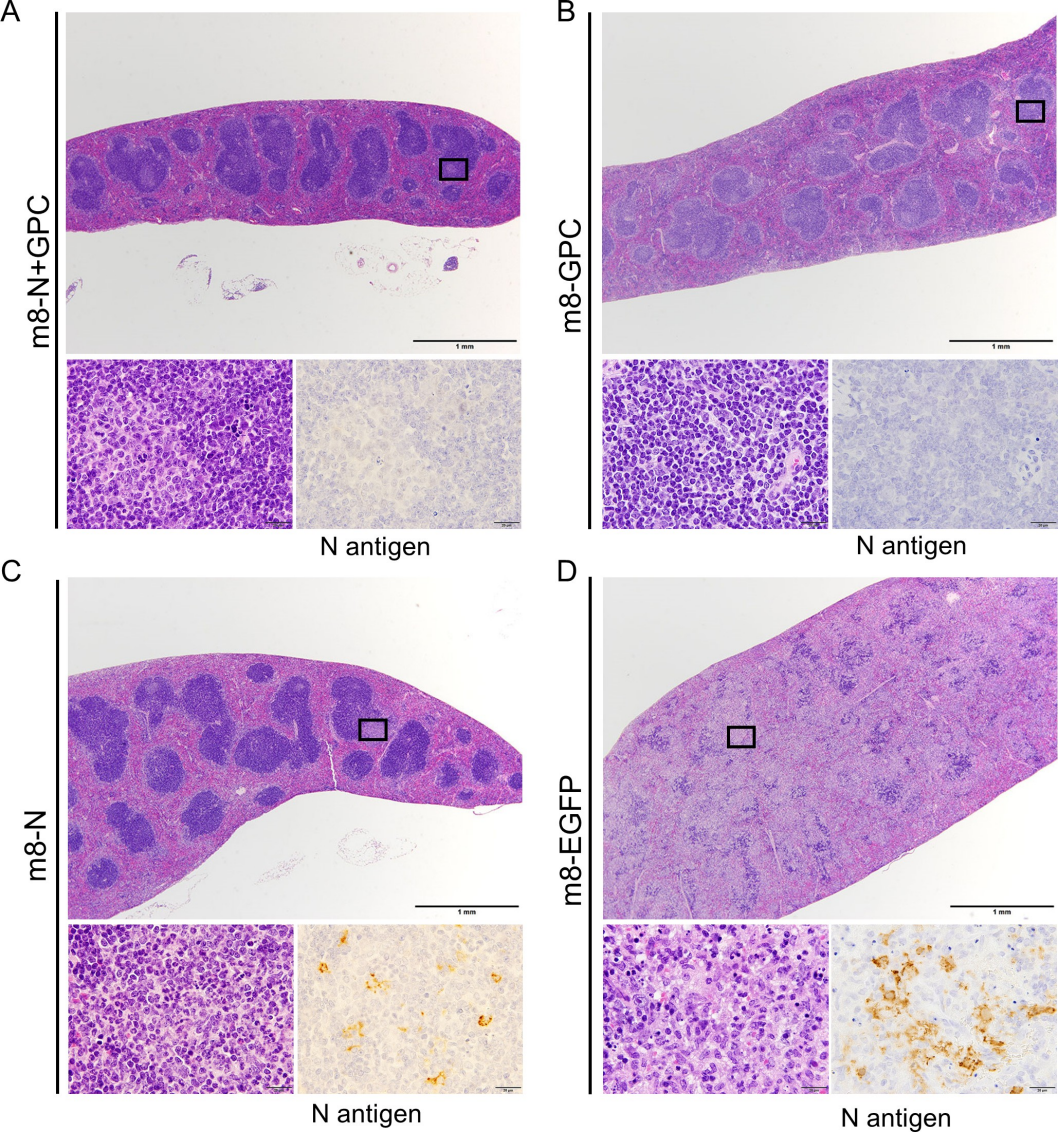
Histopathological changes in the spleen at 5 days of mice inoculated with m8-based vaccine after SFTSV challenge. Histopathology of spleens from mice inoculated with m8-N+GPC (A), m8-GPC (B), m8-N (C), and m8-EGFP (D) after SFTSV challenge was shown. High-magnification views of hematoxylin and eosin-stained tissue sections (left) and immunohistochemistry (right) of the areas of interest are shown at the bottom of the low magnification views of each group.

The liver, kidney, spleen, and cervical lymph nodes excised from mice inoculated with m8-N, m8-GPC, m8-N+GPC, and m8-EGFP after SFTSV challenge at 1, 3, and 5 DPI were subjected to histopathological and immunohistochemical analyses. The pathological changes of these organs differed among the 3 m8-based SFTSV vaccination groups and the control mice inoculated with m8-EGFP. There were no histopathological changes and any cells with viral antigen in the examined organs excised from m8-GPC or m8-N+GPC-inoculated mice (Table 1 and 2 and Fig 7A and 7B). The m8-N-inoculated mice showed focal infiltration of lymphocytes in the liver at 1 and 3 DPI, although viral antigen-positive cells were not detected at that time. At 5 DPI, there were pathological changes, including multifocal necrosis in the liver, mild interstitial lymphocytic infiltration in the kidney, focal infiltration of neutrophils in the white pulp of the spleen (Fig 7C top and bottom left), and depletion of lymphocytes in the cervical lymph nodes. Furthermore, in the m8-N-inoculated mice, viral antigen-positive large mononuclear cells were detected only spleen and cervical lymph nodes, as was also observed in m8-EGFP-inoculated mice (Table 2 and Fig 7C bottom right). This observation correlated with the finding that a higher level of viral RNA was detected in the spleen of the m8-N-inoculated mice at 5 DPI (Figs 6C and 7C). In the control mice, the m8-EGFP-inoculated mice, the following histopathological changes were observed. Focal necrosis with infiltration of neutrophils and lymphocytes and mild infiltration of mononuclear cells were detected in the liver and kidney at 1 DPI. These hepatic and renal lesions gradually spread over time (Table 1). Lymphocyte depletion with apoptosis and infiltration of neutrophils were also detected in some white pulps in spleen and cervical lymph nodes at 3 DPI, and the lesions progressed as severe lymphocyte depletion with massive necrosis in the splenic white pulps, and cervical lymph nodes caused follicular structure almost disappeared on 5 DPI (Fig 7D top and bottom left). Some viral antigen-positive cells that were large mononuclear cells began to be detected from 3DPI (Table 2 and Fig 7D bottom right), and the cells were consistently localized with the lesions, including the liver, kidney, spleen, and lymph nodes.

**Table 1:** Temporal change of lesion-positive tissues in SFTSV-challenged mice.

Vaccines inoculated	Mice with SFTSV-infection specific lesions in tissues (no. of positive/no. of mice)											
	Liver (DPI)			Spleen (DPI)			Kidney (DPI)			Cervical LN (DPI)		
	1	3	5	1	3	5	1	3	5	1	3	5
m8-EGFP	3/3	3/3	3/3	0/3	2/3	3/3	3/3	3/3	3/3	0/3	1/3	3/3
m8-N	3/3	3/3	3/3	0/3	0/3	1/3	0/3	0/3	1/3	0/3	0/3	3/3
m8-GPC	0/3	0/3	0/3	0/3	0/3	0/3	0/3	0/3	0/3	0/3	0/3	0/3
m8-N+GPC	0/3	0/3	0/3	0/3	0/3	0/3	0/3	0/3	0/3	0/3	0/3	0/3

DPI, days post infection

**Table 2:** Temporal change of SFTSV N antigen-positive tissues in SFTSV-challenged mice.

Vaccines inoculated	SFTSV N antigen-positive mice in tissues (no. of positive/no. of mice)											
	Liver (DPI)			Spleen (DPI)			Kidney (DPI)			Cervical LN (DPI)		
	1	3	5	1	3	5	1	3	5	1	3	5
m8-EGFP	0/3	2/3	3/3	0/3	3/3	3/3	0/3	1/3	3/3	0/3	2/3	3/3
m8-N	0/3	0/3	0/3	0/3	0/3	2/3	0/3	0/3	0/3	0/3	0/3	3/3
m8-GPC	0/3	0/3	0/3	0/3	0/3	0/3	0/3	0/3	0/3	0/3	0/3	0/3
m8-N+GPC	0/3	0/3	0/3	0/3	0/3	0/3	0/3	0/3	0/3	0/3	0/3	0/3

DPI, days post infection

These results indicated that vaccination with either of the m8-based SFTSV vaccines conferred protective immunity that suppressed the propagation of SFTSV and inflammation *in vivo*. However, it is noteworthy that the efficacy induced by the m8-based SFTSV GPC vaccine is superior to that induced by vaccination with m8-N.

### Contribution of antibodies induced by m8-based SFTSV vaccine

**Fig 8:**
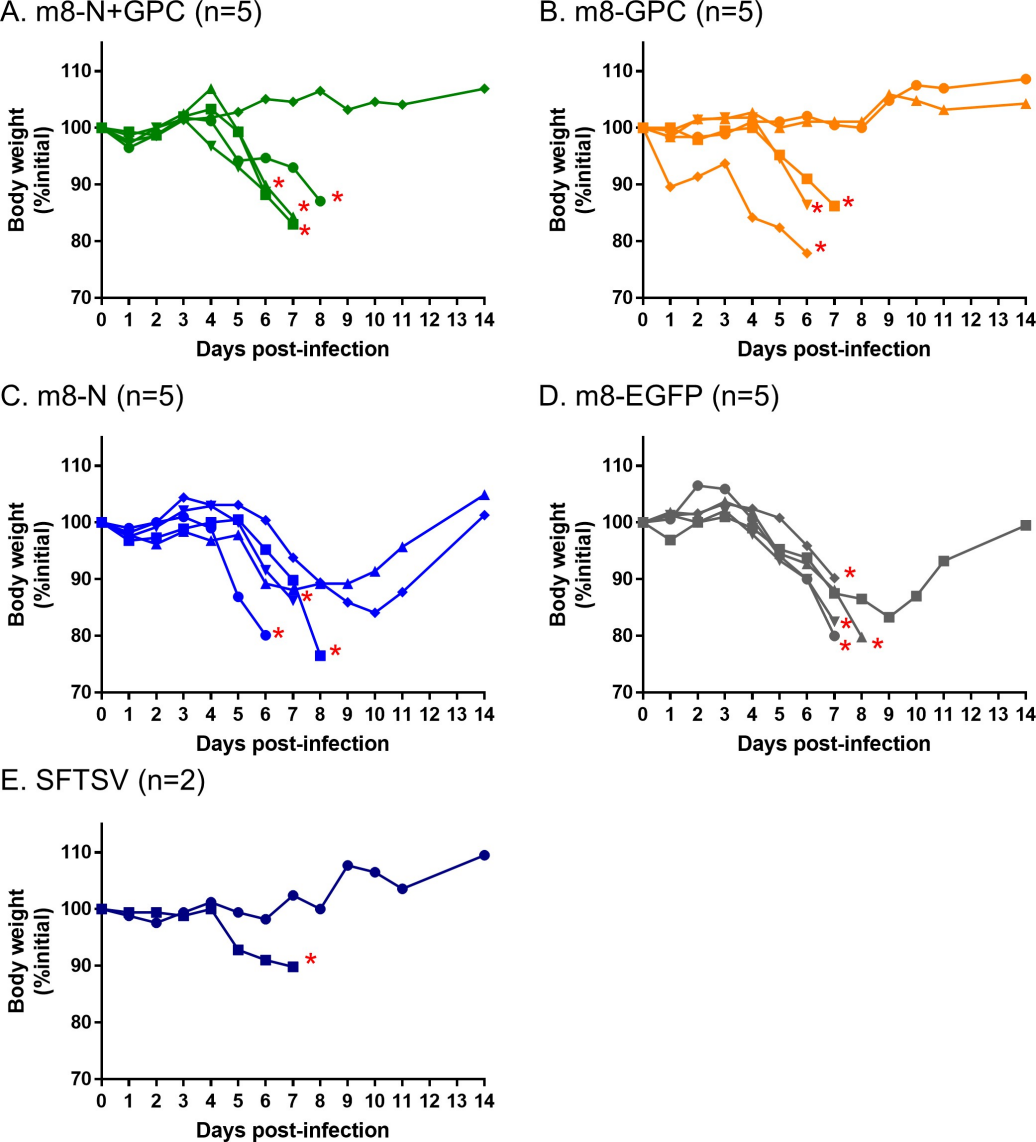
Weight change in mice to which sera collected from mice inoculated with each m8-based SFTSV vaccine was passively administered. IFNAR-/- mice were passively transferred with 100 μl of sera collected from mice inoculated m8-EGFP (A), m8-N (B), m8-GPC (C) or m8-N+GPC (D) at 1 day before and immediately before the subcutaneous challenge with SFTSV YG-1 at a dose of 1 × 10^3^ TCID_50_. As a positive control, sera obtained from mice 3 weeks after the infection with 10 TCID_50_ of SFTSV YG-1 was administered to a group of naïve mice (E). The percent weight change of each individual in the groups from the initial weight was measured daily for 2 weeks. The number of mice used in a group is shown as “n” in the legend. The asterisk indicates the endpoint of the individuals.

**Fig 9:**
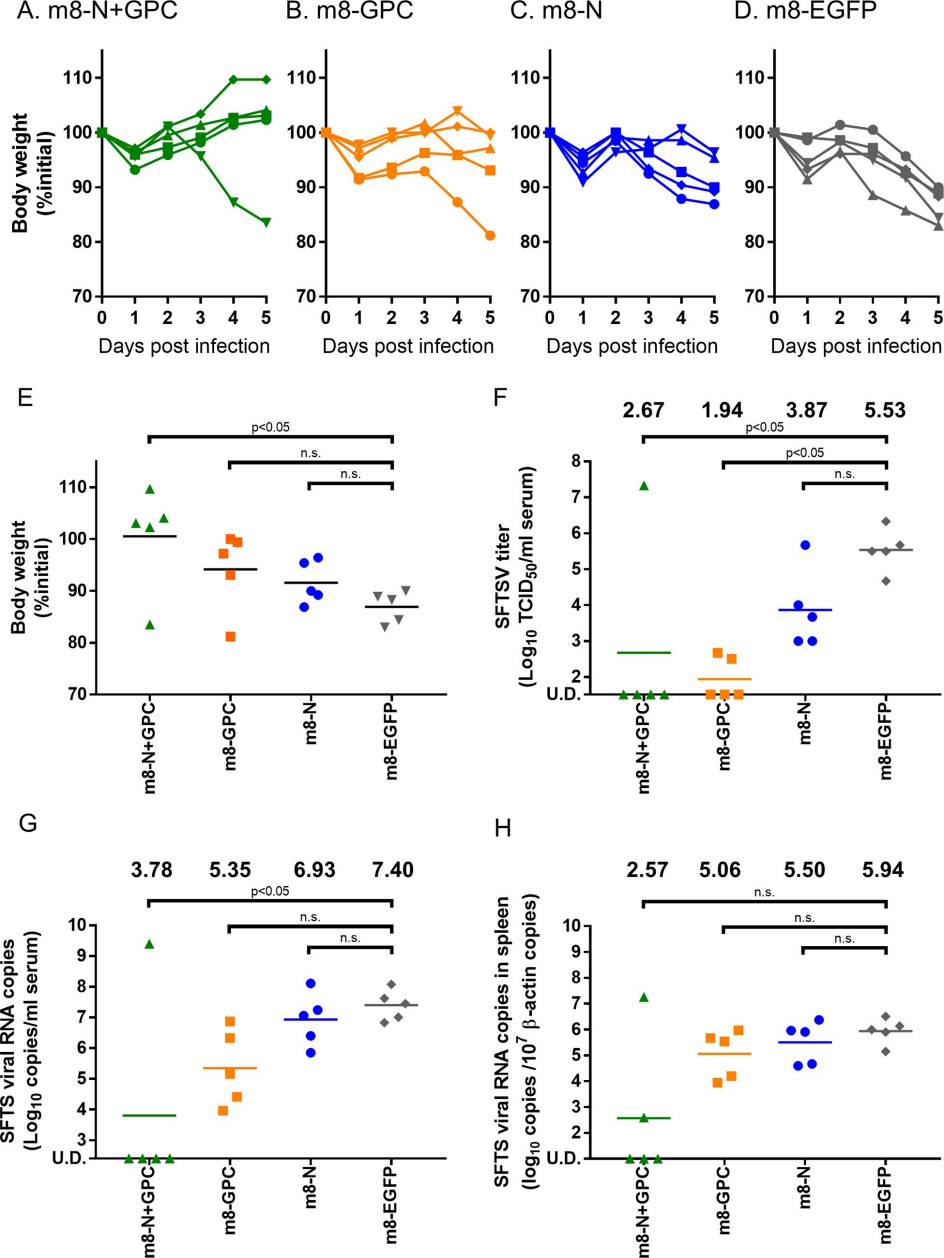
Sequential changes in body weight and viral load in mice to which sera collected from mice inoculated with each m8-based SFTSV vaccine was passively administered. IFNAR-/- mice (five per group) were passively transferred with 400 μl of pooled sera collected from mice inoculated with m8-EGFP (A), m8-N (B), m8-GPC (C), or m8-N+GPC (D) on -1, 0, and 1 DPI of the subcutaneous SFTSV challenge at 1 × 10^3^ TCID_50_. The percent weight change of each individual in each group from the initial weight was measured daily until 5 DPI. The percent weight change from the initial at 5DPI was plotted (E). The infectious SFTSV titers in the sera were measured with a standard TCID_50_ assay (F). The SFTSV RNA copies were determined with qPCR in the sera (G) and in the spleen (H). The values are shown after normalization based on the 1 × 10^7^ copies of the murine β-actin genome at 5 DPI. The mean values in each mouse group are shown at the top of each graph (F-H). When the virus titers and SFTSV RNA copy numbers were less than those of the detection limit, the infectious virus titers and the viral RNA copy numbers were defined as the detection limit (32 TCID_50_ per ml for infectious virus titers, 125 copies per ml of serum and 10 copies per 1 × 10^7^ copies of murine β-actin in the spleen for viral RNA copy numbers). Kruskal-Wallis test with Dunn’s multiple-comparison test was used to determine the level of statistical significance. The calculated p-values are shown above the groups that were compared. U.D., under the detection limit, which is 32 TCID_50_ per ml, 125 copies per ml of serum, or 10 copies per 1 × 10^7^ copies of murine β-actin in the spleen.

To verify the contribution of the humoral immunity induced by m8-based SFTSV vaccines, sera obtained from the mice inoculated with each vaccine in the previous experiment as shown in Fig 3 was passively transferred to naïve mice and challenged with SFTSV. As a control, a group of naïve mice was inoculated with sera obtained from mice three weeks after the infection with 1 × 10^1^ TCID_50_ of SFTSV. Six- to 10-week-old naïve IFNAR-/- mice (5 or 2 per group) underwent the intraperitoneal administration of 100 μl sera one day before (-1 DPI) and immediately before (0 DPI) SFTSV challenge at a dose of 1 × 10^3^ TCID_50_ subcutaneously. The sera used for this passive serum transfer experiment were those collected from the mice inoculated with each m8-based vaccine. To evaluate the efficacy of serum collected from each mouse group inoculated with each m8-based SFTSV vaccine, the association between the antibody titers in the sera and the outcome was evaluated. The individual IFA and NT antibody titers in the sera used for this serum passive transfer experiment, which are shown as a whole in Fig 3, are shown (Table 3). The survival rate of the mice treated with each serum collected from mice immunized with each m8-based SFTSV vaccine showed no significant improvement in comparison to the control of the m8-EGFP inoculated mice (Fig 8). However, there were several mice that did not develop any weight loss in each group of mice treated with sera collected from m8-GPC-, m8-N+GPC-, or YG-1-inoculated mice. There was no significant correlation between the level of individual 50% NT antibody titers and the recipient mouse’s outcome after the SFTSV challenge (Table 3). Based on the results, increased amounts of sera were used for the next passive transfer experiment. Groups of naïve IFNAR-/- mice (15 to 16 mice per group) were subcutaneously inoculated twice at a 2-week interval with each m8-based SFTSV vaccine. The sera were collected from the mice and were pooled within each mouse group. The pooled sera were confirmed to contain the viral protein-specific and NT antibodies against SFTSV (Table 4). Next, five- to six-week-old naïve IFNAR-/- mice (five per group) were administered intraperitoneally with 400 μl of pooled sera on -1, 0, and 1 DPI of SFTSV challenge. The sera and spleens were collected from the mice challenged at 5 DPI and subjected to determine infectious SFTSV titers and SFTSV RNA copy numbers. The trend of the bodyweight loss apparently improved only in the group of m8-N+GPC-mice’s sera-administered mice in comparison with that of the m8-EGFP-mice’s sera-administered mice (Fig 9A–9D). Although the body weight change seemed to be improved in the m8-GPC-mice’s sera-administered mice and the m8-N-inoculated mice’s sera-inoculated mice at 5 DPI, the statistically significant improvement was achieved only in the m8-N+GPC-mice’s sera-administered mice compared to the control of m8-EGFP-mice’s sera-administered mice (Fig 9E). The infectious SFTSV titers in serum were significantly less in the m8-N+GPC-mice’s sera-administered mice and in the m8-GPC-mice’s sera-administered mice than those of the control, the m8-EGFP-mice’s sera-administered mice (Fig 9F). Interestingly, the SFTSV titer in the sera of m8-N-mice’s sera-administered mice was 1.66 log10 times lower than that of m8-EGFP-mice’s sera-administered mice, although the difference was not statistically significant. In general, viral RNA levels measured with quantitative RT-PCR does not reflect the levels of infectious virus. Four out of five m8-N+GPC-mice’s sera-administered mice did not contain detectable amounts of SFTSV RNA, and the levels were significantly lower than the m8-EGFP-mice’s sera-administered mice (Fig 9G). The viral RNA levels in the sera collected from the m8-GPC-mice’s sera-administered mice were approximately 2.0 log10 times lower than that of m8-EGFP, although the difference was not statistically significant. Furthermore, the levels of SFTSV RNA in the spleen of m8-N+GPC-mice’s sera-administered mice were 3.37 log10 times lower than that of the m8-EGFP-mice’s sera-inoculated mice. However, the difference was not also statistically significant (Fig 9H). These results indicated that transfer of both the humoral immunity against SFTSV GPs and N simultaneously contributed—to a certain degree—to conferring anti-SFTSV protective immunity.

**Table 3:** Antibody titers in individual mice sera used for the serum passive transfer experiment and the outcome of the recipients after SFTSV challenge.

Inoculum	Mouse no.	IFA titer against SFTSV (log2)^a^		50% NT antibody titer^a^	Outcome^b^
		N	GPC		
m8-N+GPC	1	13	13	<10	Dead
	2	13	12	10	Dead
	3	13	12	40	Dead
	4	13	11	40	Dead
	5	13	11	40	Alive
m8-GPC	1	<3	12	160	Alive
	2	<3	11	<10	Dead
	3	<3	14	10	Alive
	4	<3	12	10	Dead
	5	<3	13	10	Dead
m8-N	1	13	<3	<10	Dead
	2	13	<3	<10	Dead
	3	13	<3	<10	Alive
	4	12	<3	<10	Dead
	5	14	<3	<10	Alive
m8-EGFP	1	<3	<3	<10	Dead
	2	<3	<3	<10	Alive
	3	<3	<3	<10	Dead
	4	<3	<3	<10	Dead
	5	<3	<3	<10	Dead
SFTSV	1	16	14	10	Alive
	2	15	12	40	Dead

^a^ These IFA and 50% NT antibody titers are shown in Fig 3 as well.

^b^ Outcomes of each mouse, which was passively transferred with the serum from recombinant m8s or SFTSV-inoculated mouse against SFTSV challenge are also shown in Fig 8.

### Contribution of CD8-positive cells against SFTSV infections

**Fig 10:**
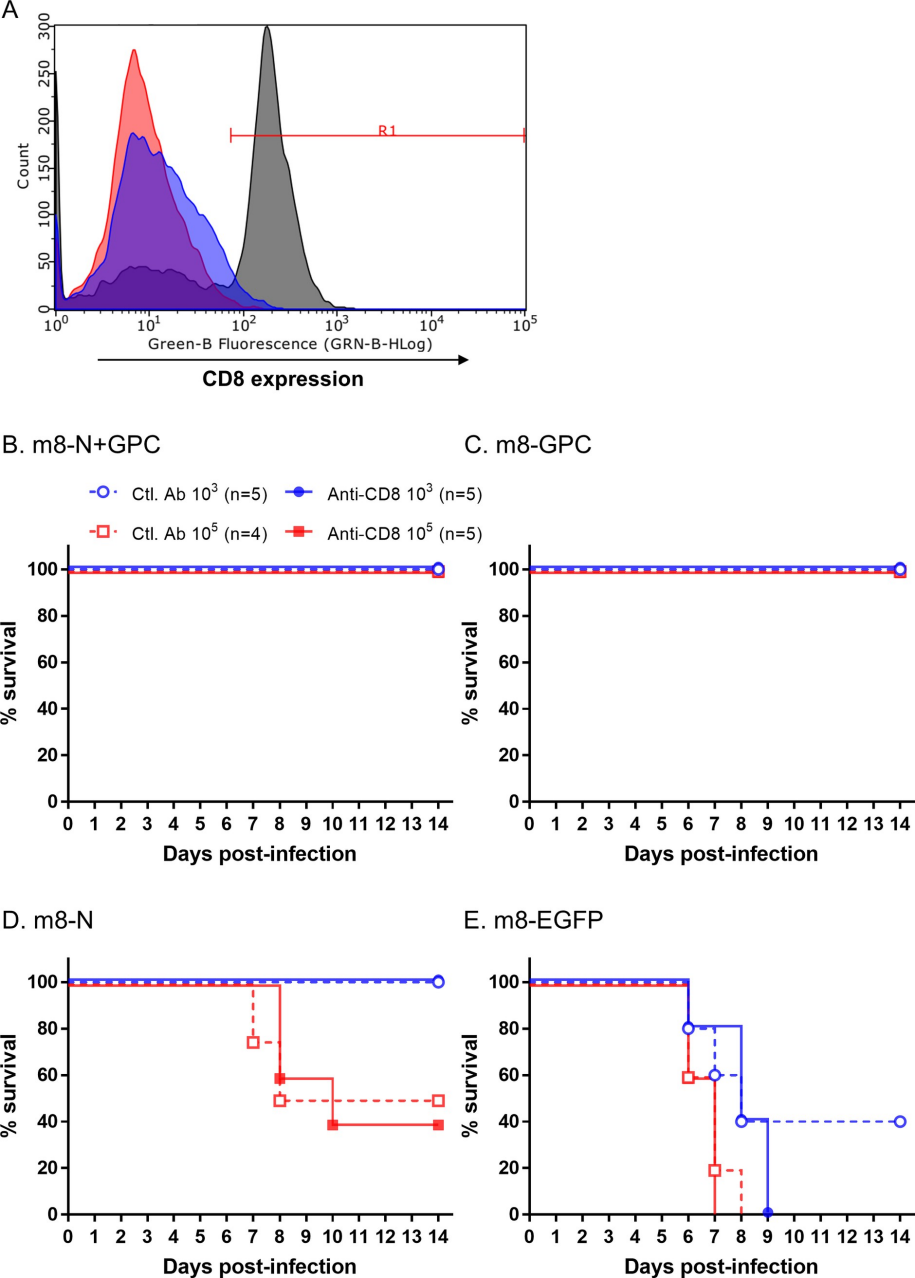
Survival in m8-SFTSV gene-inoculated mice with CD8-positive cell depletion following lethal SFTSV challenge. The efficacy of anti-CD8 mAb in depletion of CD8-positive cells in naïve IFNAR -/- mice were validated (A). A gate (R1), which separated a population of CD8 positive and negative cells, was adjusted using the histogram of splenocyte on 1 day after control mAb inoculation (Gray histogram), and the ratio of CD8 negative to positive cells was 36.1:63.9. Based on the gate, the ratio of CD8 negative:positive cells in splenocytes at 1 day (blue histogram) and 3 days (red histogram) after anti-CD8 mAb inoculation was 96.1:3.9 and 99.2:0.8, respectively. Next, IFNAR-/- mice were subcutaneously inoculated twice at a 2-week interval with a dose of 1 × 10^6^ PFU of the m8-based SFTSV vaccines. Two weeks after the second inoculation of mice with m8-N+GPC (A), m8-GPC (B), m8-N (C) or m8-EGFP (D), the mice were challenged with 1 × 10^3^ (blue circle) or 1 × 10^5^ (red square) TCID_50_ of SFTSV YG-1 and were inoculated with anti-CD8 (strait line with filled symbol) mAb, to deplete the CD8-positive cells, or control (dotted line with open symbol) mAb on -1, 2, 5, and 8 DPI before and after SFTSV challenge. Survival was evaluated daily for 2 weeks. The number of mice in a group is shown as “n” in the legend.

**Fig 11:**
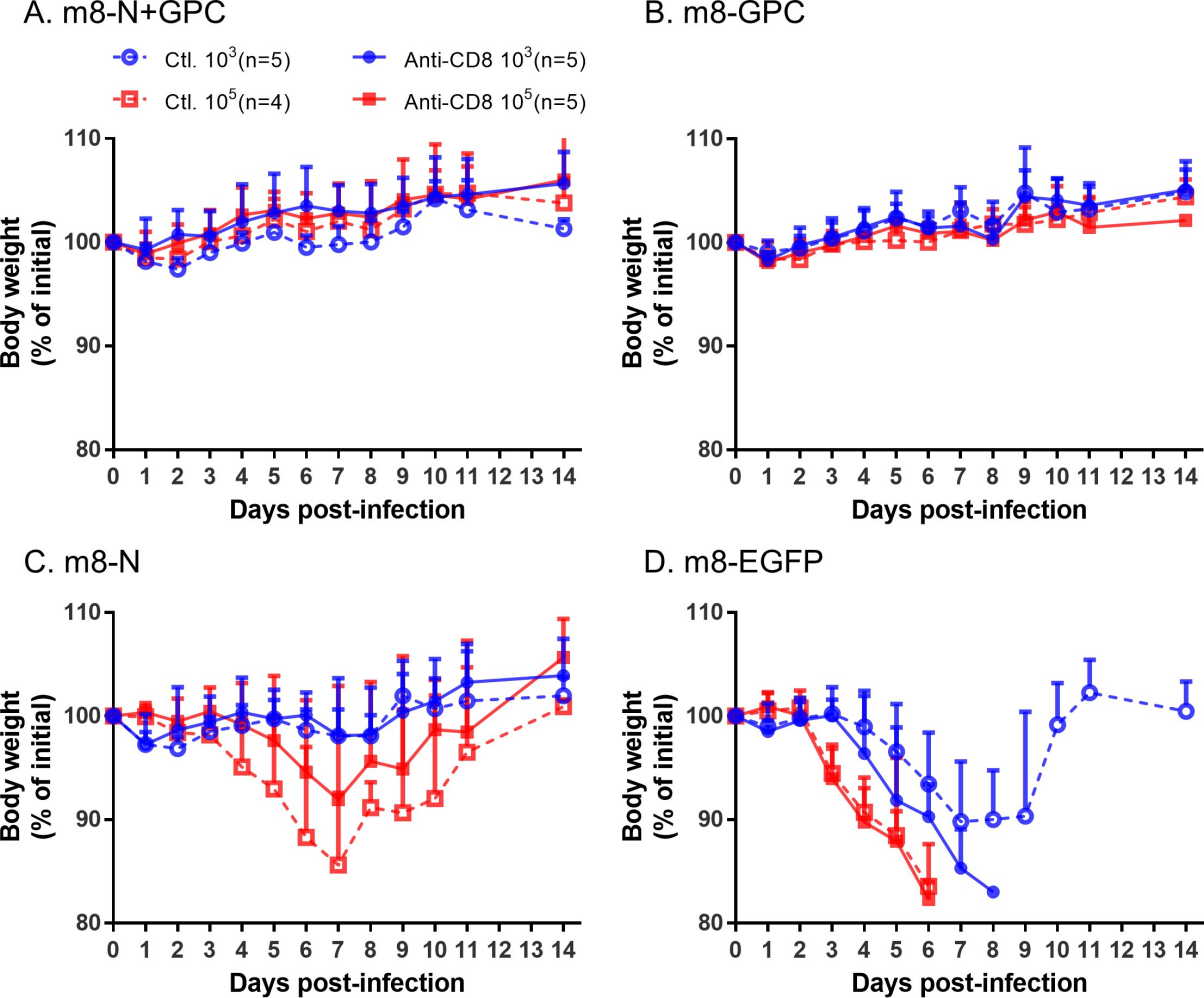
Weight change in m8-SFTSV gene-inoculated mice following a lethal SFTSV challenge with CD8-positive cell depletion. The inoculation schedule and figure legend are described in Fig 10. The percent weight change from the initial weight was measured daily for 2 weeks.

The efficacy of the anti-CD8 mAb for the depletion of CD8-positive cells *in vivo* was at first validated by using naïve IFNAR-/- mice. The population of CD8 negative:positive cells in the splenocytes collected on 1 day and 3 days after anti-CD8 mAb inoculation were 96.1:3.9 and 99.2:0.8, respectively when 250 μg of rat anti-mouse CD8 mAb clone 2.43 was intraperitoneally administered to each mouse, whereas the population in the control mice inoculated with the control mAb was 36.1:63.9 on 1 day after inoculation (Fig 10A). This result indicates that CD8-positive cells were depleted not completely but efficiently. Next, CD8-positive cells were depleted *in vivo* during SFTSV challenge, which was performed to verify the contribution of CD8-positive cells, which are known to play a significant role in cellular immunity. The depletion of CD8-positive cells during the SFTSV challenge did not alter the survival rate in mice inoculated with either of the m8-based SFTSV vaccines or in the mice inoculated with the control m8-EGFP (Fig 10B–10E). Furthermore, there was no marked difference in the sequential change in body weight in the groups treated with anti-CD8 and control mAb (Fig 11). These results suggested that CD8-positive cells did not contribute to conferring anti-SFTSV protective cellular immunity.

## Discussion

An SFTS vaccine is urgently needed. One of the strategies for developing safe and effective vaccines is to use recombinant vaccine vectors. VAC has been served as a recombinant vaccine vector for many types of infectious diseases, including highly pathogenic infectious diseases [18]. In particular, MVA-based recombinant vaccines for CCHF and Ebola virus disease were reported to be effective in animal models [27–29]. Since there are many similarities between CCHF and SFTS (e.g., *Bunyavirales* order, tick-borne infectious disease, viral hemorrhagic fever, and other clinical findings) [30, 31], we hypothesized that an m8-based recombinant vaccine might be effective for SFTS.

Interestingly, this study elucidated both m8-N and m8-GPC conferred protection against lethal SFTSV challenge in mice, although m8-N appeared less effective than m8-GPC. Whereas CCHF virus (CCHFV) GPC-expressing MVA but not CCHF virus (CCHFV) N-expressing MVA conferred protection against lethal CCHFV challenge [27, 28]. It is hypothesized that the active acquired immunity elicited by SFTSV N and CCHFV N is dominantly cellular immunity, since the specific antibody cannot access the N in the virion. Therefore, cellular immunity against SFTSV infection could play a more crucial role than that of CCHFV infection.

The expression of the GPC gene in the cells infected with the Uukuniemi virus and Rift Valley fever virus in *Phenuiviridae* is necessary and sufficient for forming the VLP [32–34]. Since VLP presents antigens densely and repetitively in general, VLP is advantageous for vaccine use as it is efficiently taken up and processed by antigen-presenting cells, and also activates B cells via the physical crosslinking of B cell receptors [35, 36]. In this study, it was confirmed that the SFTSV VLP was produced in the cells *in vitro* when infected with m8-GPC or m8-N+GPC. As for the live recombinant SFTSV vaccines, m8-GPC and m8-N+GPC have the advantage of VLP production, which might occur not only *in vitro* but also *in vivo*, eliciting strong cellular and antibody responses. Although the immunogenicity (in terms of the humoral immunity) in mice induced by authentic SFTSV infection was slightly higher than that induced by recombinant m8-based SFTSV vaccines (Fig 3), the immune response induced by vaccination with m8-GPC or m8-N+GPC completely protected against lethal SFTSV infection (Fig 4). This result suggests that the mechanism through which m8-N+GPC induced protective immunity to SFTSV infection by mimicked that of authentic SFTSV infection.

The main target population for SFTS vaccination is people over 50 years of age, since age is a critical risk factor for suffering from severe and lethal SFTS [4, 37–39]. All countries terminated the routine smallpox vaccine program for infants by 1980. For example, the routine smallpox vaccination program was terminated in Japan in 1976. Since the youngest individuals who received the smallpox vaccine are now in their 40s, preexisting immunity to VAC is a considerable factor that might affect the ability of m8-based vaccines to induce protection against SFTSV. This study demonstrated the significant efficacy of the m8-GPC and m8-N+GPC vaccine in mice pre-vaccinated with VAC, even though there was only a one-month interval between the VAC (Lister) inoculation and m8-based SFTSV vaccines inoculation (Fig 5). There have been no orthopoxvirus epidemics in the more than 40 years since people who received the smallpox vaccine received their last vaccination. Thus, it is highly likely that m8-based SFTSV vaccines will still be effective for individuals who have previously received the smallpox vaccine.

In general, recombinant VAC elicits both humoral (i.e., antibodies) and cellular immunity (i.e., cytotoxic T lymphocytes) against a target antigen [27, 28]. The key players in the humoral and cellular immunity are antibodies and cytotoxic CD8-positive T lymphocytes. In the passive serum transfer experiments, the antibodies induced by m8-N+GPC and m8-GPC significantly reduced the level of infectious SFTSV *in vivo* (Fig 9F). Still, the level of efficacy was different (Fig 9). The IFA antibody titers to SFTSV GPC and 50% NT antibody titers in the pooled sera of m8-N+GPC-inoculated mice were higher than those of m8-GPC-inoculated mice, but the difference was small (Table 4). An apparent difference was the induction ability of specific antibodies against SFTSV N between m8-N+GPC and m8-GPC. In the present study, m8-N did not induce NT antibodies to SFTSV *in vitro*. However, the transfer of the pooled sera collected from m8-N-inoculated mice reduced SFTSV titers ten times more than those collected from the m8-EGFP-inoculated mice *in vivo*, although the difference between the two groups was not statistically significant. A similar result has been reported, namely, that mice were protected from influenza virus infection, and influenza virus was cleared from the lung significantly by transferring influenza virus nucleoprotein-immune serum to the mice intraperitoneally [40]. Also, the protection was induced by an FcγR- and CD8 T cell-dependent manner. From the results, the authors speculated that FcγR-dependent activation of dendritic cells by immune complexes formed with anti-NP antibodies might enhance viral antigen-presenting activity to CD8 T cells. Although further study is necessary, it is evident that the antibodies against N could have the ability to reduce the SFTSV replication capacity *in vivo* by an unknown mechanism. Also, the immune response against SFTSV N might work additively or synergistically with that against the GPC. On the other hand, SFTSV gene-specific CD8-positive cells elicited by m8-based SFTSV vaccines were dispensable for the protection against the SFTSV challenge. It is possible that CD8-positive cells were effective but might not be depleted completely. Further studies are needed to clarify the role of cellular immunity in the protection of subjects, including humans, from SFTSV infection.

**Table 4:** Antibody titers in pooled sera used for the serum passive transfer experiments.

Inoculum	IFA titer against SFTSV (log2)		50% NT antibody titer
	N	GPC	
m8-N+GPC	13	12	40
m8-GPC	< 3	11	10
m8-N	13	< 3	< 10
m8-EGFP	< 3	< 3	< 10

Interestingly, it was previously reported that *in vivo* depletion of CD8-positive T lymphocytes in rats immunized with recombinant VAC that expresses measles genes did not alter the protective effect against a measles virus challenge [41, 42]. Although the authors did not test the *in vivo* depletion of CD4-positive cells, they hypothesized that the CD4-positive T cell-mediated immune response specific for measles gene products was sufficient to control measles virus infection. The contribution of CD4-positive T cells in mice inoculated with each of the m8-based SFTSV vaccines during the SFTSV challenge should be studied.

In summary, a novel recombinant m8 that expresses SFTSV N, GPC, or both N and GPC was generated. m8-GPC-infection and m8-N+GPC-infection of the cells induced the production of SFTSV-like-particles. In addition to m8-GPC and m8-N+GPC, m8-N was confirmed to confer protective efficacy against lethal SFTSV infection in mice. We concluded that m8-based SFTSV vaccines are promising candidates for SFTS.

## Materials and methods

### Ethics statement

All experiments associated with animals were performed in animal biological safety level 2 or 3 containment laboratories at the National Institute of Infectious Diseases in Japan (NIID) under strict regulations of the animal experimentation guidelines of the NIID. The protocol was approved by the Institutional Animal Care and Use Committee of the NIID (no. 117059, 117130, 117131, 118127, 118129 and 118173).

### Cells and viruses

293FT cells (Thermo Fisher Scientific, Waltham, MA), Vero cells, and RK13 cells were grown in Dulbecco’s Modified Eagle’s Medium (DMEM; Wako, Osaka, Japan) supplemented with 5% of heat-inactivated fetal bovine serum (FBS) and antibiotics (Sigma-Aldrich Japan, Tokyo). VAC strain Lister, which is the grandparental strain of m8 (accession No. AY678276) [43], m8 (accession No. AY678275), and recombinant m8 expressing an enhanced green fluorescent protein (m8-EGFP), which was generated previously [44] were propagated in RK13 cells. The infectious titer in RK13 cells was determined by a standard plaque-forming unit (PFU) assay [43]. SFTSV strains YG-1 (accession No. AB817979, AB817987, AB817995), SPL005 (AB817982, AB817990, AB817998), and SPL010 (AB817983, AB817991, AB817999) were propagated in Vero cells. The infectious dose of SFTSV strain YG-1 in Vero cells was determined with the 50% standard tissue culture infectious dose (TCID_50_) assay, with visualization of infection on the wells in a 96-well plate by an IFA, as described previously [14], because the CPE was not apparent for visualization as a characteristic of SFTSV infection *in vitro*. Briefly, cells infected with SFTSV were reacted with an in-house-made rabbit anti-SFTSV N polyclonal antibody, then stained with Alexa Fluor 488-conjugated goat anti-rabbit IgG H+L antibody (Thermo Fisher Scientific).

### Plasmids

The open reading frames (ORFs) of N and GPC of SFTSV strain SPL005 were amplified using RNA purified from cells infected with SFTSV by two-step conventional RT-PCR. These ORFs containing a synthetic VAC early/late promoter [45] were also cloned into the NotI (N) or an XhoI (GPC) site of precB5R, a plasmid for generating recombinant m8s by homologous recombination [44]. The plasmids inserted with N and GPC genes were named precB5R-N and precB5R-GPC, respectively. The ORF of N with the vaccinia early/late promoter was inserted into the NotI site of precB5R-GPC to construct a plasmid named precB5R-N+GPC that contained both ORFs: N and GPC. The mammalian expression plasmid pHEK293 Ultra Expression Vector II (Takara Bio Inc., Shiga, Japan) was also inserted with the N or GPC ORF at the BamHI site of multicloning sites. Plasmids with the insertion of N and GPC were named pHEK293-N and -GPC, respectively. The N protein expression plasmid was inserted with an N gene with a FLAG-tag at the C-terminus of the gene.

### Generation of recombinant m8 harboring SFTSV genes

Recombinant m8, m8-N, m8-GPC and m8-N+GPC, with the insertion of only the N gene, only the GPC gene, and both the N and GPC genes, respectively, in the flanking region between B4R and B6R genes, were generated with a method employing homologous recombination for foreign gene insertion, as described previously [44]. Briefly, 293FT cells were transfected with each of the following plasmids: precB5R-N, precB5R-GPC, and precB5R-N+GPC by using X-tremeGENE 9 (Roche Diagnostics K.K., Tokyo, Japan). Cells transfected with each plasmid were then infected with m8 at a multiplicity of infection (MOI) of 0.05 per cell. The cells were cultured in DMEM supplemented with 5% FBS for 3 days. The culture medium, along with the cells, was then collected and was freeze-thawed three times to prepare crude intermediate recombinant m8 stocks. RK13 cell monolayers were inoculated with each of the crude intermediate recombinant m8 stocks. Then, the inoculated stock was removed, and the cells were overlaid with Eagle’s Minimal Essential Medium (EMEM, Wako) containing 2% of FBS, 20 μg/ml mycophenolic acid (MPA, Sigma-Aldrich), 250 μg/ml xanthine, 15 μg/ml hypoxanthine, and 1% agarose M.E. (Iwai Chemicals, Tokyo, Japan). After three days, the mCherry fluorescence-positive plaques were identified by either fluorescence microscopy or an LED transilluminator (GELmieru, WAKO) at the wavelength of 500 nm. One fluorescence-positive plaque in the agarose plug was collected and mixed with DMEM supplemented with 5% FBS to prepare stocks of the intermediate recombinant m8 clones. RK13 cells in a monolayer in a 96-well plate were inoculated with the intermediate clones to confirm the expression capability of SFTSV N and/or GPC using an IFA. The cells inoculated were fixed with a 1:1 methanol: acetone mixture at two DPI. The cells were then reacted with either an in-house-made rabbit anti-SFTSV N polyclonal antibody or an in-house mouse anti-SFTSV Gc mAb clone C6C1, followed by reaction with DyLight 594-conjugated goat anti-rabbit IgG H+L antibody (Abcam, Cambridge, UK) and DyLight 488-conjugated goat anti-mouse IgG H+L antibody (Abcam), respectively. The fluorescence-positive expression clones were then further purified by plaque isolation at least three times. RK13 cells were infected with the purified intermediate clones without supplementation with MPA, xanthine, and hypoxanthine to prepare the crude final recombinant m8 stocks. Final recombinant m8-based SFTSV vaccines, from which the selection marker genes were self-excised, resulting in their deletion, were cloned by selecting fluorescence-negative plaques. Hence, monolayered RK13 cells were infected with one of the final crude recombinant m8 stocks, and the cells were overlaid with agarose containing MEM supplemented with 2% FBS without supplementation with MPA, xanthine, and hypoxanthine. After three days, the mCherry fluorescence-negative plaques were collected under an LED transilluminator. The N and/or GPC expression-positive and the mCherry fluorescence-negative clones were next purified by plaque isolation at least three times to establish the final recombinants. The SFTSV genes expression in cells infected with each final recombinant virus was confirmed using the IFA. Briefly, the cells on a tissue culture plate were infected with m8-N, m8-GPC, m8-N+GPC, or m8-EGFP, and the cells were then fixed with a 1:1 methanol:acetone mixture, which quenched the EGFP fluorescence, at 3 DPI. Since the vaccinia virus spreads via cell-to-cell transmission, the plaques formed without overlaying medium (e.g., agarose or methylcellulose) to prevent fluid movement in the culture. The cells were reacted with a mixture of an in-house-made rabbit anti-SFTSV N polyclonal antibody and an in-house mouse anti-SFTSV Gc mAb, followed by a reaction with a mixture of DyLight 594-conjugated goat anti-rabbit IgG H+L antibody (Abcam) and DyLight 488-conjugated goat anti-mouse IgG H+L antibody (Abcam). The m8-N, m8-GPC, m8-N+GPC, and m8-EGFP were propagated in RK13 cells, and their infectious doses were determined with the standard plaque assay.

### Confirmation of SFTS virus-like-particles (VLP)

RK13 cells were infected with m8-GPC or m8-N+GPC at an MOI of 0.1. The culture supernatant was collected at 3 DPI and mixed with polyethylene glycol (PEG) 6000 at a final concentration of 8 w/v % for pelleting the VLP, and the mixture was incubated at 4°C overnight. The PEG precipitates were pelleted by centrifugation for 30 min at 1,500 ×g at 4°C in a swing-out rotor. The pellets were resuspended with DMEM supplemented with 5% FBS. The VLP was purified through a double-cushion of 20% and 60% sucrose in H_2_O in an SW-41 swing-out rotor by ultracentrifugation (Beckman Coulter, Brea, CA) for 2 hours at 25,000 rpm at 4°C in a swing-out rotor. The accumulated VLP that was banded at the 20/60% interface was negatively stained with 2% phosphotungstic acid and then observed using a JEM-1400 transmission electron microscope (JEOL, Tokyo, Japan).

### Immunoprecipitation

The incorporation of SFTSV N, Gn, or Gc into the VLP was confirmed by immunoprecipitation. Briefly, RK13 cells were infected with m8-EGFP, m8-N, m8-GPC, or m8-N+GPC at an MOI of 0.05. The culture supernatants were collected to evaluate the N and the GPs incorporations into the VLP at 2 DPI and 3 DPI, respectively. The remaining cells were lysed with sodium dodecyl sulfate (SDS) sample buffer and heated at 95°C for 5 min. One milliliter of each supernatant was incubated with 15 μl of goat anti-mouse IgG magnetic beads (New England Biolabs, Ipswich, MA) with rotation overnight at 4 ^o^C, following the addition of 3 μg of mouse anti-SFTSV Gc mAb (clone C6C1) or not. The magnetic beads in the supernatant were collected on a magnetic stand and washed 2 times with PBS. Twenty microliters of SDS sample buffer was added to the beads and heated at 95°C for 5 min. The infected cell lysates and immunoprecipitated samples were fractionated by SDS polyacrylamide gel electrophoresis (SDS-PAGE) and subjected to western blotting. The membrane was reacted with rabbit anti-SFTSV N polyclonal antibody, rabbit anti-SFTSV Gn antibody (number NBP2-41153, Novus biologicals), rabbit anti-SFTSV Gc antibody (number NBP2-41156, Novus biologicals, Centennial, CO) or rabbit anti-GAPDH polyclonal antibody (number 10494-1-AP, Proteintech, Rosemont, IL), followed by reaction with horseradish peroxidase (HRP)-conjugated goat anti-rabbit IgG (H+L) antibody (KPL, Gaithersburg, MD). The immunoreactive proteins were visualized by LAS-4000mini (Fujifilm, Tokyo, Japan) with ECL Prime Western blotting detection reagents (G.E. Healthcare, Chicago, IL). The N or GP proteins density on the western blots were measured by using ImageJ Fiji 1.53c [46].

### Animals

IFNAR-/- mice on the C57BL/6 background were used for all of the animal experiments. The IFNAR-/- mice were previously produced by mating DNase II/type I interferon receptor double-knockout mice (strain B6.129-Dnase2a<tm1Osa> Ifnar1<tm1Agt>) [47–49] and C57BL/6 mice (age, 4–10 weeks; sex, male or female) and bred under specific pathogen-free (SPF) conditions at the NIID [50].

### Evaluation of vaccine efficacy in VAC-immunonegative mice

The mice were subcutaneously inoculated twice at a two-week interval with 1 × 10^6^ PFU of m8-EGFP, m8-N, m8-GPC, or m8-N+GPC in a volume of 100 μl per mouse under anesthesia with midazolam, medetomidine and butorphanol tartrate combination. At two weeks after the second inoculation, the mice were euthanized with Isoflurane and sacrificed to collect blood to evaluate immunogenicity or subcutaneously challenged with 1 × 10^3^ or 1 × 10^5^ TCID_50_ of SFTSV strain YG-1 in a volume of 100 μl per mouse under anesthesia with midazolam, medetomidine and butorphanol tartrate combination to evaluate the protective efficacy of each m8-based SFTSV vaccine candidate. The body weight and clinical signs of the mice challenged with SFTSV was monitored daily for two weeks. In the experiments challenged with SFTSV, the mice were euthanized with Isoflurane when the mice became moribund or expected to be die since of the difficulty in eating and/or drinking.

### Evaluation of vaccine efficacy in VAC-immunopositive mice

Four- to five-week-old IFNAR-/- mice (10 per group) were subcutaneously inoculated with 1 × 10^6^ PFU of a VAC strain Lister, a second-generation smallpox vaccine strain, in a volume of 100 μl per mouse at 4 weeks prior to the first inoculation with the m8-based SFTSV vaccine. The VAC pre-inoculated mice were then subcutaneously inoculated twice at a two-week interval with each m8-based SFTSV vaccine followed by subcutaneous challenge with 1 × 10^3^ or 1 × 10^5^ TCID_50_ of SFTSV YG-1, as described above. The body weight and clinical signs of the mice challenged with SFTSV was monitored daily for two weeks.

### Validation of the efficacy of anti-CD8 mAb in CD8-positive cells depletion *in vivo*

Naïve mice were inoculated intraperitoneally with 250 μg of rat anti-CD8 mAb clone 2.43 (BioXCell, West Lebanon, NH) to deplete CD8 positive cells *in vivo*, as described previously [51]. Rat anti-keyhole limpet hemocyanin mAb clone LTF-2 (BioXCell) was also used as the control antibody. The spleen was collected on 1 day or 3 days post antibody administration. The spleen cells were resuspended in PBS with 2% fetal calf serum (FCS) and stained with FITC conjugated anti-CD8a mAb (clone 53–6.7, Thermo Fisher Scientific). Flow cytometry data were acquired on a Guava easyCyte Flow Cytometers (Luminex, Austin, TX).

### Evaluation of the anti-SFTSV antibody transfer efficacy and cellular immunity in mice

The following experiment was designed to evaluate the humoral immunity induced by vaccination with m8-based SFTSV vaccines. Sera were collected from mice subcutaneously inoculated twice with 1 × 10^6^ PFU of m8-EGFP, m8-N, m8-GPC, or m8-N+GPC at a two-week interval, as described above. Naïve mice were passively transferred twice with 100 μl of sera at 1 day (-1 DPI) and just before (0 DPI) the subcutaneous SFTSV challenge at 1 × 10^3^ TCID_50_. In another experiment, naïve mice were passively transferred three times with 400 μl of pooled serum mixture collected from each mouse group inoculated with 1 × 10^6^ PFU of m8-EGFP, m8-N, m8-GPC, or m8-N+GPC on -1, 0, and 1 DPI of the subcutaneous SFTSV challenge at 1 × 10^3^ TCID_50_. To evaluate the cellular immunity induced by m8-based SFTSV vaccines, CD8-positive cells were depleted *in vivo* by the administration of anti-CD8 mAb. The 6- to 9-week-old IFNAR-/- mice (4 to 5 per group) were subcutaneously inoculated twice with 1 × 10^6^ PFU of m8-EGFP, m8-N, m8-GPC, or m8-N+GPC at a 2-week interval, as described above. Two weeks later, the mice were subcutaneously challenged with 1 × 10^3^ or 1 × 10^5^ TCID_50_ of SFTSV YG-1 and were intraperitoneally inoculated with 250 μg of anti-CD8 mAb clone 2.43 or Rat anti-keyhole limpet hemocyanin mAb clone LTF-2 as the control on -1, 2, 5, and 8 DPI from SFTSV challenge. The body weight and clinical signs of the mice challenged with SFTSV was monitored daily for two weeks.

### Indirect immunofluorescence assay

IFA antigen-spotted slides, 293FT cells were transfected with either pHEK293-N or pHEK293-GPC with an expression enhancer plasmid, pHEK293 (Takara Bio Inc.). A mixture of the 293FT cells transfected with expression vectors in combination and untransfected 293FT cells at a ratio of 1:3 were washed with PBS, spotted on glass slides (Matsunami glass IND., Ltd., Osaka, Japan), dry-fixed, and treated with acetone. To measure the titer of the SFTSV N- or GPC-specific IgG in mice, serum samples immobilized at 56°C for 30 min were serially diluted two-fold and added onto the antigens on the glass slides. The antigens were then reacted with an Alexa Fluor 488-conjugated goat anti-mouse IgG H+L antibody (Thermo Fisher Scientific). The antibody titer was defined as the reciprocal of the highest dilution level at which a specific fluorescent signal was detected.

### Focus reduction neutralization test

To measure the titer of neutralizing antibody to SFTSV in mouse serum, 100 TCID_50_ aliquots of SFTSV were mixed with 4-fold serially diluted immobilized sera, and the mixtures were incubated for 1 hour. Confluent Vero cell cultures in 12-well microtiter plates were inoculated with each virus and serum mixture for 1 hour, overlaid with DMEM containing 2% FBS and 1% methylcellulose, and then cultured for 4 to 5 days. The cells were fixed with PBS containing 10% formalin and were reacted with rabbit anti-SFTSV N polyclonal antibody. The foci of SFTSV infection were then visualized using a standard immunoperoxidase method. The percent NT antibody titer was determined to divide the number of foci in the well inoculated with the virus and serum from m8-SFTSV gene or SFTSV inoculated mouse by that of naïve mice. The 50% NT antibody titer was defined as the reciprocal of the serum dilution level resulting in a 50% reduction of foci.

### Quantitative one-step RT-PCR

Specific quantitative one-step reverse transcription-PCR (qPCR) was performed for measuring the copy numbers of SFTSV RNA as reported previously [52]. Specific qPCR primer and probe sets targeted to the SFTSV genome S segment (forward; TGTCAGAGTGGTCCAGGATT, reverse; ACCTGTCTCCTTCAGCTTCT, probe; FAM-TGGAGTTTGGTGAGCAGCAGC-BHQ1) and murine β-actin (forward; TGCTGACAGGATGCAGAAGG, reverse; ACTCCTGCTTGCTGATCCAC, probe; FAM-TCGGTGGCTCCATCCTGGCC-BHQ1) were used. Total RNAs were extracted from 200 μl of mice sera or spleens using a High Pure viral RNA kit (Roche Applied Science) or ISOGEN with spin column kit (NIPPON GENE, Tokyo, Japan), respectively, according to the manufacturer’s protocols. The elution volume for RNA extraction was 50 μl. For the qPCR assay, an aliquot of the extracted RNA solution was added to the reaction mixture for the QuantiTect probe RT-PCR kit (Qiagen, Hilden, Germany), which contained 2x QuantiTect probe RT-PCR master mix, QuantiTect RT mix, H2O, and 10x primer-probe mix, which contained 4 μM of each specific primer, 2 μM TaqMan probe(s), and 2 μM of contamplicon probe. The reverse transcription reaction was carried out at 50°C for 30 min, followed by 45 cycles of amplification under the following conditions: 94°C for 15 s and 60°C for 60 s after PCR activation at 95°C for 15 min in a LightCycler 96 (Roche).

### Histopathology and immunohistochemistry

As described above, IFNAR-/- mice were subcutaneously inoculated with 1 × 10^6^ PFU of the m8-EGFP or each m8-based SFTSV vaccine twice at a 2-week interval and were intraperitoneally challenged with 1 × 10^5^ TCID_50_ of SFTSV YG-1, 2 weeks after the second inoculation. The SFTSV-infected mice were euthanized with Isoflurane and sacrificed at 1, 3, and 5 DPI, and the blood, cervical lymph nodes, spleen, liver, and kidneys were collected. The tissues were fixed with PBS containing 10% formalin and embedded with paraffin to prepare blocks. Sections were prepared from the tissue block and were stained with hematoxylin-eosin for a histopathological examination. An immunohistochemical analysis to detect SFTSV N protein was performed as previously described [14]. The sections were first reacted with a rabbit anti-SFTSV N polyclonal antibody. Antigens were retrieved by hydrolytic autoclaving in citrate buffer (pH 6.0) for 10 min at 121°C. IHC staining was then performed using a standard immunoperoxidase method.

### Statistical analysis

All statistical analyses were performed using GraphPad Prism 7 (GraphPad Software, La Jolla, CA). Survival curves were plotted according to a Kaplan-Meier analysis, and the protective efficacy of the m8-based SFTSV vaccines against SFTSV challenge in mice was evaluated using a log-rank test with Bonferroni correction to adjust the significance level for multiple comparisons. The equality of the median of the 50% NT antibody titers in mouse sera, the median of N protein density on the western blots, body weight change, SFTSV titers in the sera and SFTSV RNA copies in sera and spleens were evaluated using a Kruskal-Wallis test with Dunn’s multiple-comparison test. The equalities of the means of the infectious virus titers and the viral RNA levels in mouse sera were evaluated using a two-way analysis of variance (ANOVA) with Sidak’s multiple-comparison test. P values of <0.05 were considered to indicate statistical significance.

## Supporting information

S1 Fig Confirmation of SFTS N protein incorporation into the VLP. The experimental details and figure legend are described in Fig 1.
(TIF)
