# Supplementary figures and images for: A highly attenuated vaccinia virus strain LC16m8-based vaccine for severe fever with thrombocytopenia syndrome
Source: PLoS Pathog. 2021 Feb 3;17(2):e1008859. doi: 10.1371/journal.ppat.1008859 (PMC7886154; doi:10.1371/journal.ppat.1008859)

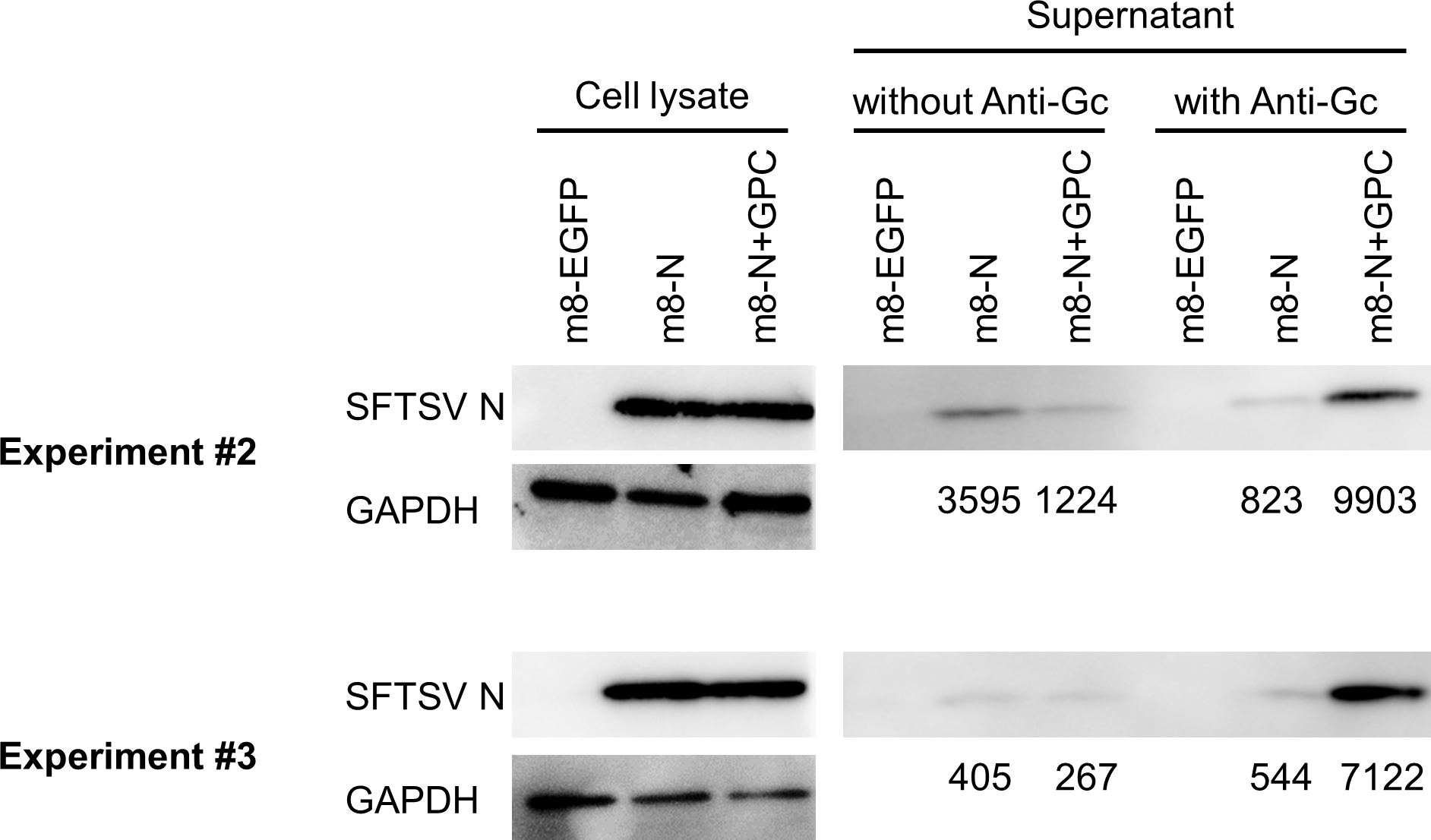

Supplement: S1 Fig — The experimental details and figure legend are described in Fig 1. (TIF) [file ppat.1008859.s001.tif]
